# Epithelial Expressed B7-H4 Drives Differential Immunotherapy Response in Murine and Human Breast Cancer

**DOI:** 10.1158/2767-9764.CRC-23-0468

**Published:** 2024-04-24

**Authors:** Elizabeth C. Wescott, Xiaopeng Sun, Paula Gonzalez-Ericsson, Ann Hanna, Brandie C. Taylor, Violeta Sanchez, Juliana Bronzini, Susan R. Opalenik, Melinda E. Sanders, Julia Wulfkuhle, Rosa I. Gallagher, Henry Gomez, Claudine Isaacs, Vijaya Bharti, John T. Wilson, Tarah J. Ballinger, Cesar A. Santa-Maria, Payal D. Shah, Elizabeth C. Dees, Brian D. Lehmann, Vandana G. Abramson, Gillian L. Hirst, Lamorna Brown Swigart, Laura J. van ˈt Veer, Laura J. Esserman, Emanuel F. Petricoin, Jennifer A. Pietenpol, Justin M. Balko

**Affiliations:** 1 Department of Pathology, Microbiology, and Immunology, Vanderbilt University Medical Center, Nashville, Tennessee.; 2 Breast Cancer Research Program, Vanderbilt-Ingram Cancer Center, Vanderbilt University Medical Center, Nashville, Tennessee.; 3 Department of Medicine, Vanderbilt University Medical Center, Nashville, Tennessee.; 4 Department of Biological Sciences, Vanderbilt University, Nashville, Tennessee.; 5 Center for Applied Proteomics and Molecular Medicine, George Mason University, Manassas, Virginia.; 6 Department of Medical Oncology, Instituto Nacional de Enfermedades Neoplásicas, Lima, Perú.; 7 Division of Hematology-Oncology, Department of Medicine, Georgetown University, Washington, District of Columbia.; 8 Department of Chemical and Biomolecular Engineering, Vanderbilt University, Nashville, Tennessee.; 9 Division of Hematology and Oncology, Indiana University School of Medicine, Indianapolis, Indiana.; 10 Johns Hopkins Sidney Kimmel Comprehensive Cancer Center, Baltimore, Maryland.; 11 Abramson Cancer Center, University of Pennsylvania, Philadelphia, Pennsylvania.; 12 Department of Medicine, School of Medicine, University of North Carolina, Chapel Hill, North Carolina.; 13 Department of Surgery, University of California San Francisco, San Francisco, California.; 14 Department of Laboratory Medicine, University of California San Francisco, San Francisco, California.; 15 Department of Biochemistry, Vanderbilt University Medical Center, Nashville, Tennessee.; 16 Cancer Biology Program, Vanderbilt University, Nashville, Tennessee.

## Abstract

Combinations of immune checkpoint inhibitors (ICI, including anti-PD-1/PD-L1) and chemotherapy have been FDA approved for metastatic and early-stage triple-negative breast cancer (TNBC), but most patients do not benefit. B7-H4 is a B7 family ligand with proposed immunosuppressive functions being explored as a cancer immunotherapy target and may be associated with anti-PD-L1 resistance. However, little is known about its regulation and effect on immune cell function in breast cancers. We assessed murine and human breast cancer cells to identify regulation mechanisms of B7-H4 *in vitro.* We used an immunocompetent anti-PD-L1–sensitive orthotopic mammary cancer model and induced ectopic expression of B7-H4. We assessed therapy response and transcriptional changes at baseline and under treatment with anti-PD-L1. We observed B7-H4 was highly associated with epithelial cell status and transcription factors and found to be regulated by PI3K activity. EMT6 tumors with cell-surface B7-H4 expression were more resistant to immunotherapy. In addition, tumor-infiltrating immune cells had reduced immune activation signaling based on transcriptomic analysis. Paradoxically, in human breast cancer, B7-H4 expression was associated with survival benefit for patients with metastatic TNBC treated with carboplatin plus anti-PD-L1 and was associated with no change in response or survival for patients with early breast cancer receiving chemotherapy plus anti-PD-1. While B7-H4 induces tumor resistance to anti-PD-L1 in murine models, there are alternative mechanisms of signaling and function in human cancers. In addition, the strong correlation of B7-H4 to epithelial cell markers suggests a potential regulatory mechanism of B7-H4 independent of PD-L1.

**Significance:**

This translational study confirms the association of B7-H4 expression with a cold immune microenvironment in breast cancer and offers preclinical studies demonstrating a potential role for B7-H4 in suppressing response to checkpoint therapy. However, analysis of two clinical trials with checkpoint inhibitors in the early and metastatic settings argue against B7-H4 as being a mechanism of clinical resistance to checkpoints, with clear implications for its candidacy as a therapeutic target.

## Introduction

Immune checkpoint inhibitors (ICI), including anti-PD-1/anti-PD-L1 mAbs, have become a staple in the clinical treatment of many cancer types (1). The immune system is highly regulated to promote protective responses against pathogens and cancer, while also inhibiting adverse inflammation and autoimmunity. Effector lymphocytes of the immune system therefore express immunosuppressive proteins, such as CTLA-4 and PD-1, that bind to their respective ligands, CD80/86 and PD-L1 to inhibit exacerbated inflammation. Cancer cells and other cells in the tumor microenvironment can express T-cell coinhibitory molecules of the B7 family, like PD-L1, to evade or suppress adaptive immunity. Together, these proteins work to downregulate inflammation and induce an immunosuppressive environment in tumors (2–5).

Many types of immune cells infiltrate tumors, including T cells and myeloid cells, to induce a proinflammatory, antitumor response. However, tumor cells or other microenvironmental cells expressing PD-L1 or other coinhibitory ligands, can engage infiltrating T cells and suppress T-cell activation (3, 5). Therefore, infiltrating lymphocytes in PD-L1^+^ tumors are likely unable to eradicate the tumor. Currently approved ICIs target the immune system by preventing inhibitory interactions between these suppressive cells and infiltrating lymphocytes, to reinvigorate a proinflammatory response (5, 6).

ICI has seen broad success in several cancer types, including breast cancer (7, 8). Breast cancer remains one of the leading causes of new cancer diagnoses and cancer-related deaths (9). Furthermore, triple-negative breast cancer (TNBC) is one of the more difficult subtypes to treat and as such is a candidate for novel cancer therapies, like immunotherapy (10). Patients with TNBC have been the focus of immunotherapy treatment due to abundant tumor infiltrating immune cells, high tumor mutation burden, and their lack of target-specific therapies compared with other breast cancer subtypes. Many patients have had favorable outcomes with anti-PD-1/anti-PD-L1 modalities (7, 11–17). As such, pembrolizumab is now approved in combination with neoadjuvant chemotherapy (NAC) in early-stage, high-risk TNBC regardless of PD-L1 status, and in combination with chemotherapy patients with metastatic PD-L1^+^ TNBC tumors (7, 12, 16, 18–21). However, a gap in knowledge persists in predicting those patients most likely to respond to ICI therapy.

There are very few usable clinical biomarkers to identify responders versus nonresponders. For example, many breast cancer cells or infiltrating immune cells do not express PD-L1, and those that do still fail to respond to PD-1/PD-L1–targeted ICI. Because immune evasion is a hallmark of cancer, this suggests the action of alternative inhibitory pathways in many breast cancers and the potential to identify additional tumor biomarkers to predict response to ICI (22). One potential mechanism of resistance is the presence of additional immune checkpoint ligands that may override the PD-1/L1 pathway.

B7-H4 (encoded by *VTCN1*) is an immune checkpoint ligand in the CD28/B7 family of molecules characterized by sequence similarity to other B7 family proteins and is expressed in several human tumor types, including breast cancer (23–30). Several studies have suggested that B7-H4 has a coinhibitory role on tumor lymphocytes (23, 25, 29, 31–33). Its receptor has not yet been identified but is thought to be expressed on activated, but not resting, T lymphocytes (31). B7-H4 expression is associated with “immune cold” TNBC tumors that lack infiltrating and activated immune cells and is correlated with worse patient outcome (25, 26, 29, 34, 35). In contrast, PD-L1 is often expressed on highly immunogenic tumors (23, 36–38). Furthermore, published literature has shown an inverse correlation between breast tumors expressing B7-H4 and PD-L1, though no mechanism for this reciprocal pattern has been established (23, 25, 39). We sought to understand the expression and regulation of B7-H4 in breast cancers to determine whether it could be a mechanism of immune suppression and therefore a mechanism of resistance to current immunotherapies.

## Materials and Methods

### Patient Samples

Clinical specimens used for characterization of B7-H4 expression were surgically resected tumor samples collected retrospectively from 77 patients with TNBC and residual disease after NAC, diagnosed and treated at the Instituto Nacional de Enfermedades Neoplásicas under INEN 10-018 and 348 patients with ER^+^HER2^−^ and TNBC diagnosed and treated at Vanderbilt University Medical Center (VUMC) under NCT00899301 and NCT00651976. We assessed B7-H4 correlation with survival in 91 patients [with B7H4 multiplexed immunofluorescence (mIF) data] from the TBCRC 043 clinical trial (ref. 40; NCT03206203) and in 151 patients (with reverse phase protein array, RPPA, data) in the I-SPY2 clinical trial (ref. 18; NCT01042379). For TBCRC043 trial 106 patients with metastatic TNBC were randomized into two groups receiving carboplatin or carboplatin + atezolizumab; however, only 91 had viable samples for biomarker analysis of B7-H4 and were included in the analysis. For the I-SPY2 trial dataset, 151 patients were assessed that had accompanying RPPA expression data and randomized into paclitaxel control treatment (*n* = 85) or paclitaxel + pembrolizumab treatment (*n* = 66). Of the 151 patients, 62 were HR-negative and 89 were HR-positive (HR^+^).

### Cell Lines and Tissue Culture

Murine mammary cancer cell lines EMT6 and E0771 from female mice were obtained from ATCC. EMT6 cells were grown in DMEM/F12 (Gibco) supplemented with 10% FBS (Life Technologies). Murine B7-H4+ cell lines were generated using retroviral transduction with the pBabe-puro plasmid (Addgene). Positive cells were collected by FACS to obtain a pure positive population. Cell expression was regularly validated by flow cytometry.

Human female breast cancer cell line MDA-MB-468 (DMEM + 10% FBS) was obtained from ATCC. MMTV-neu cells (DMEM-F12 + 10% FBS + EGF 20 ng/mL + Hydrocortisone 0.5 µg/mL + Insulin 10 µg/mL) were derived from a spontaneous tumor within a female FVB/N-Tg (MMTV-neu) 202 Mul/J mouse. Cells were treated with 50 nmol/L trametinib (SelleckChem) or 1 µmol/L buparlisib (SelleckChem).

All cells were routinely tested (at least once quarterly and before animal injection) for *Mycoplasma* contamination using the e-Myco Mycoplasma PCR Detection Kit (LiliF Diagnostics). All media components were purchased from commercial vendors and prepared/stored under sterile conditions. Cell lines are utilized within the early passage (<30 passages from acquisition from ATCC) and are DNA fingerprinted through commercial services for validation.

### Viral Transduction

Murine B7-H4 (Genecopoeia) was cloned into pBabe-puro (Addgene) vector by restriction digest. Retroviral particles were produced by transfecting Phoenix packaging cells using Lipofectamine 3000 (Life Technologies). Target cells were transduced in the presence of polybrene and selected by puromycin resistance. pBabe-puro was a gift from Hartmut Land & Jay Morgenstern & Bob Weinberg (Addgene plasmid #1764; http://n2t.net/addgene:1764; RRID:Addgene_1764; ref. 41).

### Immunoblotting

Cells were lysed in 1 × RIPA buffer (0.1% SDS detergent, 50 mmol/L Tris pH 7.4, 150 mmol/L NaCl, 1.0% NP-40, 0.5% deoxycholic acid, 1 mmol/L Ethylenediaminetetraacetic acid (EDTA), 1 mmol/L egtazic acid (EGTA), 5 mmol/L sodium pyrophosphate, 50 mmol/L NaF, 10 mmol/L b-glycerophosphate) with added phosphatase inhibitors (PhosSTOP, Roche) and protease inhibitors (cOmplete, Roche). Lysates were sonicated and incubated on ice for 15 minutes before centrifugation at 13,000 × *g* for 10 minutes at 4°C. Protein concentrations of the lysates were determined by bicinchoninic acid assay (Thermo Fisher Scientific). Samples were separated on NuPage 4%-12% BisTris gels (Invitrogen) and transferred to nitrocellulose membranes. Membranes were blocked with 5% nonfat dry milk or 5% BSA in TBS with 0.1% Tween-20 for 1 hour at room temperature and then incubated overnight at 4°C with the appropriate antibody in blocking buffer as indicated. Following incubation with appropriate horseradish peroxidase–conjugated secondary antibodies, proteins were visualized using an enhanced chemiluminescence detection system (Thermo Fisher Scientific). This study was performed using the following antibodies: Vinculin from Santa Cruz Biotechnology (#73614), and ERK1/2 (#9102), p-ERK1/2 (#4370), AKT (#2920), p-AKT (#4060), and B7-H4 (#14572) all of which were purchased from Cell Signaling Technology.

### Flow Cytometry

Cancer cells were washed in PBS and harvested with TrypLE (Life Technologies) for 10 minutes at 37°C. Dissociated cells were washed once in PBS and incubated with respective flow antibodies at 4°C for 20 minutes in the dark for surface staining and 30 minutes for intracellular staining. Flow cytometry of cancer cells was performed using the following antibodies: B7-H4 (BioLegend #103132, 1:100 dilution), EpCAM (BioLegend #118216,1:2,000 dilution), and CD44 (BioLegend #103028, 1:1,500 dilution). Flow cytometry of tumor dissociates was performed using the following antibodies: CD45 (BioLegend #109822), TCRB (Invitrogen #48-5961-82), CD8 (Invitrogen #MA5-16759), FOXP3 (Invitrogen #12-5775-82), CD44 (BioLegend #103036), PD-1 (BioLegend #135241), Granzyme B (Invitrogen #35-8898-82), Nkp46 (BioLegend #137637), CD11b (BioLegend #101263), F4/80 (BioLegend #123120), CD206 (BioLegend #141721), Arg1 (Invitrogen #12-3697-82), and Nos2 (Invitrogen #58-5920-82). Zombie Violet (Thermo Fisher Scientific) or dye EF780 (eBioscience #65-0865-14) was used as viability dyes for dead cell exclusion. Samples were analyzed on an Attune NxT flow cytometer (Life Technologies) or CyTek Aurora and analyzed by FlowJo Version 10.

### Mice

All mice were housed at the VUMC vivarium, which is accredited by the Association for Assessment and Accreditation of Laboratory Animal Care International. Mouse procedures and studies were approved by the Vanderbilt Division of Animal Care and Institutional Animal Care and Use Committee. C57BL/6J and BALB/c mice were purchased from Envigo and allowed to acclimatize for at least 1 week before tumor implantation and experimentation. For all experiments, 6 to 8 weeks old female mice with 100–200 mm^3^ tumors were stratified into specific treatment groups.

### Tumor Implantation and Treatment Strategy

For mammary tumor models, 5 × 10^4^ EMT6 were orthotopically injected into the fourth left mammary fat pad of female BALB/c mice. Cells were tested for *Mycoplasma* contamination prior to each experiment using the e-Myco Mycoplasma PCR Detection Kit (LiliF Diagnostics). Following the establishment of tumors (∼100–200 mm^3^), mice were stratified prior to therapy administration. Mice were treated via intraperitoneal injection with isotype IgG1 control (BioXcell, clone BE0083) or anti-PD-L1 (Genentech, clone 6E11) dosing at 200 µg for the first treatment and 100 µg for two subsequent treatments at 1-week intervals. For tumor growth analysis, tumors were measured two to three times weekly with calipers, and volume was calculated in mm^3^ using the formula (length × width × width/2). Mice were humanely euthanized at defined endpoints or when the tumor volume reached 2,000 mm^3^ or tumor ulceration.

### Tumor Dissociation and Immune Cell Isolation

EMT6 tumors were harvested from mice at either 500 mm^3^ or 1-week post-treatment as indicated in figure legends and dissociated using the Mouse Tumor Dissociation Kit (Miltenyi Biotec) according to manufacturer's specifications with the gentleMACs Octo dissociator (Miltenyi Biotec) default tumor protocol (40 minutes at 37°C under constant agitation). The dissociate was then passed through a 70 µm filter, washed with 20–30 mL of PBS, and lysed using ammonium-chloride-potassium (ACK) buffer. The single cell suspension was then subjected immediately to antibody staining for flow cytometry as described above, or cell sorting by magnetic bead isolation. Dead cells were excluded using the Dead Cell Removal Kit (Miltenyi Biotec). Additional cell isolation was performed using CD45 [tumor-infiltrating lymphocyte (TIL)] mouse microbeads (Miltenyi Biotec).

### RNA Isolation

After dissociation and CD45^+^ cell isolation, RNA was harvested from mouse tumor immune cells using the Maxwell 16 automated workstation (Promega) and the LEV simplyRNA Cells Kit (Promega). RNA concentration was determined by spectrophotometry (NanoDrop2000, Thermo Fisher Scientific).

### NanoString Gene Expression Analysis

Gene expression profiles of tumor-infiltrating immune cells from either untreated or anti-PD-L1–treated B7-H4^+^ or parental EMT6 tumors were assessed using the NanoString Mouse Pan-cancer Immunology panel (770 genes) according to the manufacturer's specifications. CD45 TIL bead sorted tumor dissociates were used for RNA preparation, and 100 ng of total RNA was used for input into nCounter hybridizations. Raw RCC files were processed using NanoString nSolver to generate data frame for further data analysis. The raw count data were first batch corrected using ComBat-Seq (PMID: 33015620). Low-quality genes or samples were further filtered using negative control beads and a normalization factor is created using positive control bead and housekeeping genes to normalize the entire dataset. After normalization, the data were log transformed. Principal component analysis was performed to observe general clustering pattern and ensure no strong batch effect is present. Differential gene expression analysis was performed using Wilcox test with multiple-test correction *P* value generated. Function gene sets were directly obtained from NanoString mouse Pan-cancer immunology panel. Gene set score is calculated using a z-score sum of all the genes within the set.

### Single-cell RNA Sequencing

MMTV-neu cells were harvested directly from cell culture and prepared for single-cell RNA sequencing. Each sample (targeting 15,000 cells per sample) was processed for single-cell 5′ RNA sequencing utilizing the 10x Chromium system. Libraries were prepared following the manufacturer's protocol. The libraries were sequenced using NovaSeq 6000 with 150 bp paired-end reads. RTA (v.2.4.11; Illumina) was used for base calling. Data were analyzed in R using the filtered h5 gene matrices in the Seurat package. In brief, samples were subset to include cells with >200 but <3,000 unique transcripts to exclude probable noncellular RNA reads and doublets. Cells with >15% of reads coming from mitochondrial transcripts were also excluded as probable dying cells. Normalization, scaling, dimensional reduction, and unsupervised clustering were also performed using Seurat. Cells were classified as mesenchymal or epithelial based on *Epcam* expression.

### IHC and mIF

Formalin-fixed paraffin-embedded tissue sections were cut at 4 µm and deparaffinized. Antigen retrieval was performed with citrate buffer pH 6. Endogenous peroxidase was blocked and protein block was applied. Sections were then incubated with the primary antibodies (B7-H4 AF2154 R&D Systems at 1:600, CD45 ab10558 Abcam at 1:2,500, B7-H4 D1M8I Cell Signaling Technology 1:200, pan-Cytokeratin AE1/AE3 Biocare at 1:600, EpCAM ab71916 Abcam 1:500, CD44 ab157107 Abcam 1:1,000, CD8 144B Statlab) overnight at 4°C. For chromogenic IHC, visualization system was Envision (Agilent Technologies), diaminobenzidine (DAB) as the chromogen (Agilent Technologies) and hematoxylin was applied as the counterstain. For multiplex fluorescence IHC, sections were then incubated with the secondary antibody and tyramide signal amplifcation (TSA) reagent applied according to manufacturer's recommendations in a cyclic manner. Breast cancer with known B7-H4 expression was used as a positive control.

### Image Analysis and Quantification

Whole slide images were digitally acquired using an AxioScan Z1 slide scanner (Carl Zeiss) at 20x. Automated quantification was performed via pathologist-supervised machine learning algorithm using QuPath software (42). Tumor areas were manually annotated to exclude extensive necrosis present in most samples. For chromogenic IHC, color deconvolution to separate hematoxylin and DAB. Cell segmentation was determined on the hematoxylin. Positive cell detection algorithm according to the cell DAB optical density (OD) mean was used to calculate percent of positive tumor cells and H-score. For fluorescence IHC, cell segmentation was determined on DAPI. Object classifiers were trained on annotated training regions from control tissue and tumor samples to define cellular phenotypes. Single-cell data including sample ID, xy coordinate, cell phenotype, and B7-H4 intensity were exported from QuPath to calculate B7-H4 intensity for each cell phenotype in R.

### RPPA

RPPA was performed as described previously (43–45). Briefly, lysates were prepared and printed in triplicate spots (∼10 nL per spot) onto nitrocellulose coated slides (Grace Biolabs) using a Quanterix 2470 Arrayer (Quanterix). Standard curves of control cell lysates were included for quality assurance purposes. Antibodies used on the RPPA were validated before use by confirming the presence of a single band at the appropriate molecular weight with a panel of control cell lysates using conventional Western blotting. Immunostaining was performed by probing each slide with one primary antibody targeting the protein of interest. Biotinylated goat anti-rabbit IgG (H+L; 1:7,500, Vector Laboratories Inc) or rabbit anti-mouse IgG (1:10, DakoCytomation) were used as secondary antibodies. Signal amplification was performed using a tyramide-based avidin/biotin amplification system (DakoCytomation) followed by streptavidin-conjugated IRDye 680 (LI-COR) for visualization. Negative controls were stained with secondary antibody alone. Total protein was measured using Sypro Ruby protein blot staining per manufacturer's instructions (Molecular Probes). RPPA data were generated directly from images acquired using a Tecan PowerScanner (Tecan) and analyzed with MicroVigene software Version 5.1.0.0 (Vigenetech). Total protein intensities for each sample were calculated by averaging the Sypro staining intensity of the three replicate spots. For each sample/endpoint, the final signal intensity was calculated by: (i) subtraction of negative control spot intensity from primary antibody spot intensity, (ii) averaging the resulting net intensities for the three replicate spots, and (iii) dividing by the total protein intensity value for each sample. For the current study, anti-human-B7-H4 (clone D1M8I) XP from Cell Signaling Technology (#14572) was used.

### Data Availability

The data generated in this study are available within the article and its Supplementary Data files or are available upon request from the corresponding author. Expression data analyzed in this study were obtained from the cBioPortal Cancer Cell Line Encyclopedia (CCLE) from the Broad Institute and Novartis, updated 2019 (46). Data from the clinical trial, NCT03206203, will be available at the Sequence Read Archive (SRA) database under extension ID: PRJNA995589. Single-cell RNA sequencing data have been deposited in NCBI's Gene Expression Omnibus (GEO; ref. 47) and are accessible through GEO Series accession number GSE262654. NanoString gene expression data are available as a Supplementary File.

### Human Research Ethics

All studies using human tissues or human subjects were conducted in accordance with the Declaration of Helsinki and were performed after approval by an Institutional Review Board (IRB) and in accordance with an assurance filed with and approved by the U.S. Department of Health and Human Services, when required. The protocol for NCT03206203 was approved by ethical and IRBs (IRB#160633) at the participating institutions, and all patients provided written informed consent and did not receive financial compensation. Patients eligible for NCT01042379 are women ages 18 years or older, with stage II or III breast cancer and primary tumors larger than 2.5 cm by clinical examination or larger than 2.0 cm by imaging. All patients provide written informed consent prior to screening and again after randomization. Exceptions to this requirement were made by the IRB in cases where human tissues were part of tissue microarrays and were completely deidentified.

## Results

### B7-H4 is Expressed in Immunologically Cold Breast Tumors

**FIGURE 1:**
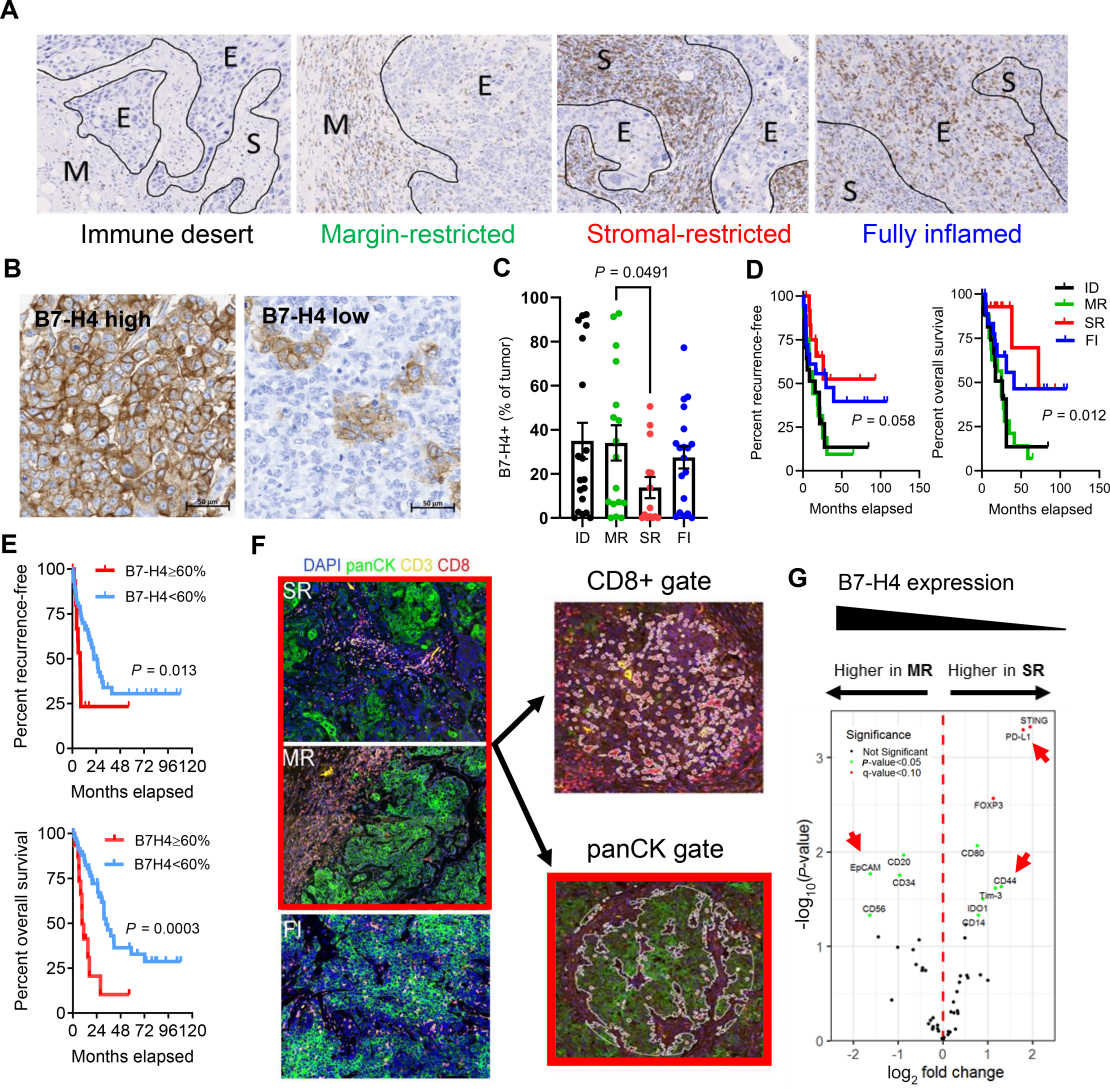
B7-H4 is associated with immune cold tumors and correlated with worse outcomes. **A,** Representative TNBC samples (residual disease, post-NAC) categorized on the basis of CD8 T-cell infiltration and localization into ID, MR, SR, and FI. **B,** B7-H4 is heterogeneously expressed in TNBC. **C,** ID and MR tumors have the highest level of B7-H4 expression (*n* = 69: ID: 19, MR: 17, SR: 14, FI: 19). MR and SR tumors were analyzed by unpaired *t* test. **D,** MR and ID tumors also have worse RFS and OS (*n* = 69). Data were analyzed by Mantel–Cox test. **E,** B7-H4 expression directly correlates with worse outcomes in patients (*n* = 77; >60%: 16, <60%: 51). Data were analyzed by Mantel–Cox test. **F,** TNBC samples stained with immunofluorescent markers for DAPI, panCK, CD3, and CD8 to identify regions of interest (ROI) for NanoString GeoMX DSP. MR and SR panCK+ ROIs were selected and differential protein expression between tumor samples is shown. **G,** Within the panCK gated cells, MR tumors had higher EpCAM expression. SR tumors had higher CD44 expression, as well as higher PD-L1. Data shown are log_2_ fold change of differentially expressed genes (*P* < 0.05, *q* < 0.10, data analyzed using R).

B7-H4 has been associated with immunologically cold tumors in contrast to PD-L1, which is often expressed in immunologically hot tumors (25, 35, 39). We sought to confirm this prior finding and characterized TNBC samples post-NAC with residual disease according to the distribution of infiltrating CD8^+^ T cells. Four groups—immune desert (ID), margin-restricted (MR), stromal-restricted (SR), and fully inflamed (FI)—were defined according to previously published metrics (refs. 25, 48; Fig. 1A). The ID and MR tumors (those exhibiting the most immunologically cold phenotypes and associated with worse outcomes) had the highest level of tumor B7-H4 expression (Fig. 1B and C), though in contrast to prior findings (25, 49), B7-H4 was also present in FI tumors, possibly due to the selective or direct molecular effects of NAC in this cohort. As has been previously shown, FI and SR tumors demonstrated improved outcomes after surgery (Fig. 1D). Regardless of microenvironment type, B7-H4 expression was associated with worse recurrence-free (RFS) and overall survival (OS) in these post-NAC TNBC samples (Fig. 1E).

Although B7-H4 has been shown to be associated with more immunologically cold tumors, other cancer cell features associated with MR and B7-H4 status have not been evaluated at the protein level. Thus, we chose MR (B7-H4 high) and SR (B7-H4 low) tumors, as well as FI tumors, and evaluated the protein expression of immune markers in the CD8^+^/cytotoxic T cell and pan-cytokeratin/tumor cell compartments using NanoString GeoMX Digital Spatial Profiling (DSP). Samples were stained with a mIF panel containing pan-cytokeratin (panCK), CD3, CD8, and DAPI to distinguish tumor cell and T-cell regions (Fig. 1F). Gating for the CD3^+^ and panCK^+^ compartments was used to extract detection antibody barcodes specifically in these cells. We compared protein expression in tumor cell regions only in MR and SR samples; ID tumors were not evaluated because they contain insufficient immune content, and FI tumors were excluded from the analysis as the dispersion of immune cells in the tumor-rich regions limited specificity of the intended gating procedure (i.e., the juxtaposition of immune cells and tumor cells limited interpretability). Although B7-H4 was not a validated detection marker in the GeoMX panel, B7-H4 expression in the tumor cells was independently validated by IHC (Fig. 1B and C). The SR samples had higher PD-L1 which was expected because of greater infiltrating immune cells and a more inflammatory microenvironment (Fig. 1G). Interestingly, these samples also had higher upregulation of the mesenchymal marker CD44 compared with MR tumors, which had higher EpCAM (epithelial status) expression (Fig. 1G). As our MR tumors had the higher B7-H4 expression, we explored further correlations between epithelial cell markers and B7-H4 expression in breast cancer.

### B7-H4 Expression is Associated with Epithelial versus Mesenchymal Status

**FIGURE 2:**
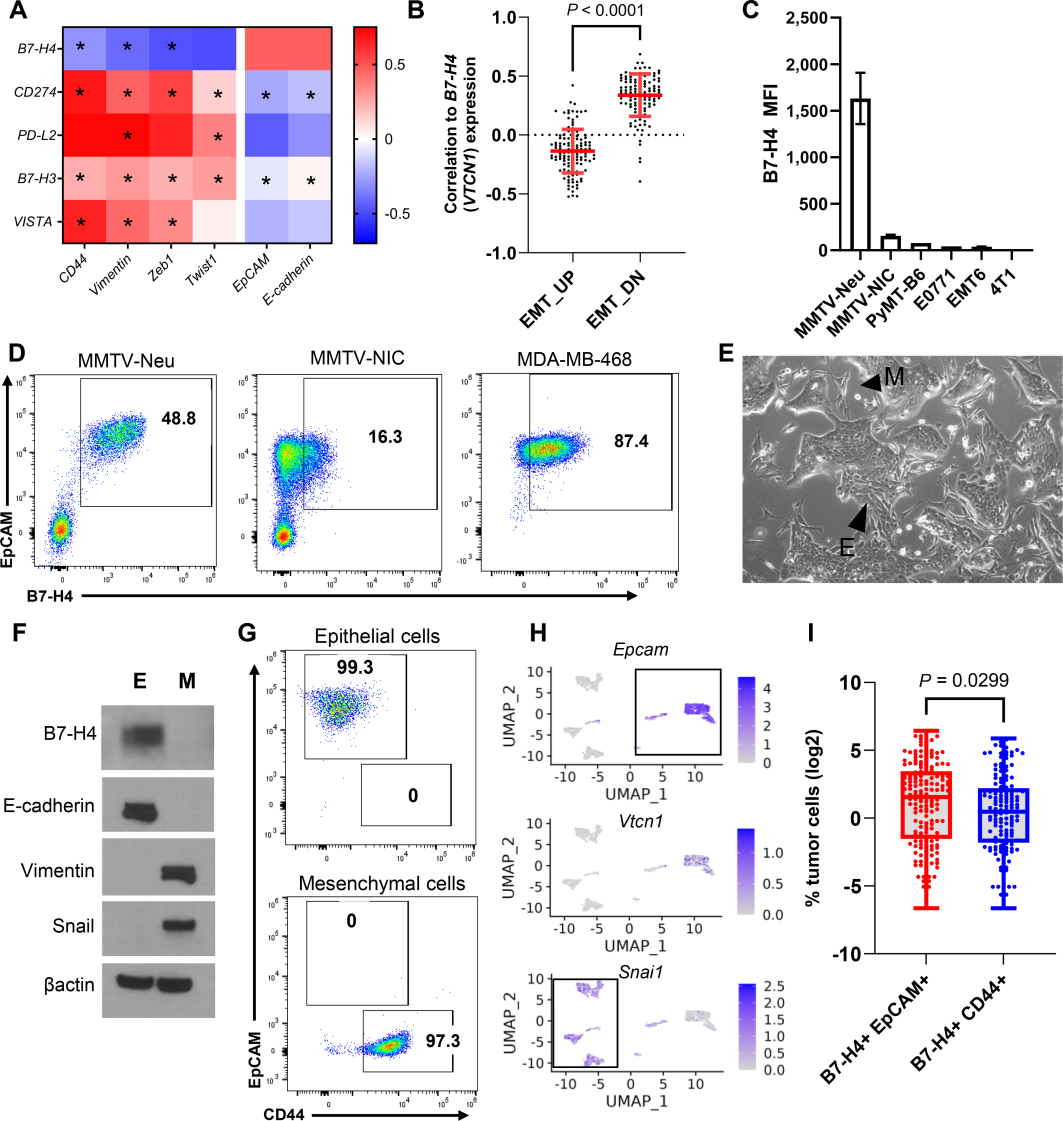
B7-H4 (*VTCN1*) is highly correlated with epithelial gene markers in mouse and human cells unlike other checkpoint ligands. **A,** In human breast cancer cell lines (CCLE), B7-H4 is the only checkpoint ligand positively correlated with epithelial markers and negatively correlated with mesenchymal markers. Data shown are Spearman correlations between genes. Significant correlations (*P* < 0.05) are indicated by asterisk. **B,** Genes downregulated during EMT are positively correlated with VTCN1 in human cell lines (CCLE). **C,** Murine cancer cell lines express B7-H4. **D,** B7-H4 is only expressed on epithelial EpCAM^+^ cells in murine and human cells. **E,** The MMTV-neu cell line with highest B7-H4 levels is comprised of phenotypically epithelial-like (abbreviated E) and mesenchymal-like (M) cells. **F,** In MMTV-neu cells, the CD44^+^ M cells do not express B7-H4 but all the EpCAM^+^ E cells do. **G,** MMTV-neu E and M cells express hallmark markers. **H,** Single-cell RNA sequencing of the heterogenous MMTV-neu cells confirms Vtcn1 is solely expressed in the epithelial cell population. **I,** A cohort of human tumors including both TNBC and ER^+^HER2^−^ were stained for B7-H4, EpCAM, and CD44 by mIF. Data shown are log_2_ of % B7-H4^+^ tumor cells and include 132 samples with >1% B7-H4 expression (paired *t* test of transformed data).

We identified over 60 breast cancer cell lines from the CCLE (50, 51) and observed strong positive correlations with markers of epithelial cell status and B7-H4 (Fig. 2A). Interestingly, other checkpoint ligands of the B7 family had an inverse relationship and were strongly associated with markers of mesenchymal cell status (Fig. 2A). In addition, epithelial-to-mesenchymal transition (EMT) transcription factors correlated with lower levels of *VTCN1* expression in these same cell lines (Fig. 2B). *VTCN1* expression was higher when EMT-associated genes had low expression and vice versa (Fig. 2B). These data suggest that B7-H4 is associated with, and could be regulated by, EMT in tumors.

We next screened several murine and human cell lines to identify a model of B7-H4 expression and perform perturbations to understand the mechanism of expression (Fig. 2C and D). The MMTV-neu mammary tumor cell line had the highest B7-H4 expression (Fig. 2C) and we used this cell line for future experiments (52, 53). This cell line, derived from an *FVB/n* mammary tumor, consists of epithelial-like and mesenchymal-like cell populations when assessed by protein and RNA expression (Fig. 2E–H). We established these cells were distinct and not actively undergoing EMT (Supplementary Fig. S1). Interestingly, the epithelial cells alone maintained B7-H4 expression (Fig. 2F and G). In one additional murine cell line (MMTV-NIC, also derived from an FVB/n mammary tumor) and the human MDA-MB-468s, epithelial cells (EpCAM^+^) expressed B7-H4 (Fig. 2D). We also performed single-cell RNA sequencing on the heterogeneous MMTV-neu cell line and observed *Epcam* expression correlated with *Vtcn1* expression on the single cell level, but *Vtcn1* was not coexpressed with *Snail1*, a mesenchymal marker (Fig. 2H). To validate the identified association of B7-H4 in epithelial cancer cells in human tumors, we stained primary ER^+^HER2^−^ and TNBC tumors for B7-H4, EpCAM, and CD44 by mIF. Once again, B7-H4 was more frequently coexpressed with EpCAM on tumor cells compared to CD44 on tumor cells (Fig. 2I). In summary, we established B7-H4 as a preferential marker of epithelial cell status, rather than mesenchymal cell status.

### B7-H4 is Regulated by PI3K Signaling in Cancer Cells

As previously stated, B7-H4 and PD-L1 expression is often mutually exclusively in breast tumors suggesting a different mechanism of regulation (23, 25, 39). We sought to identify a mechanism for differential B7-H4 expression. Because PD-L1 is highly inducible with both alpha and gamma IFN, it has been suggested that B7-H4 is similarly inducible by alpha and/or gamma IFN (28, 54–57). Conversely, we tested whether IFNs inhibited B7-H4 expression to explain the phenomenon of PD-L1 and B7-H4 mutually exclusive expression (56, 57). Treatment of MMTV-neu B7-H4^+^ cells with alpha or gamma IFN did not alter endogenous B7-H4 levels, nor was B7-H4 induced on several B7-H4–negative murine cell lines (Supplementary Fig. S2). We also tested whether TGFβ, a potent stimulator of EMT, modulated B7-H4 cell surface expression (58). We saw no change in B7-H4 expression in negative or positive cell lines by treatment with TGFβ (Supplementary Fig. S2).

**FIGURE 3:**
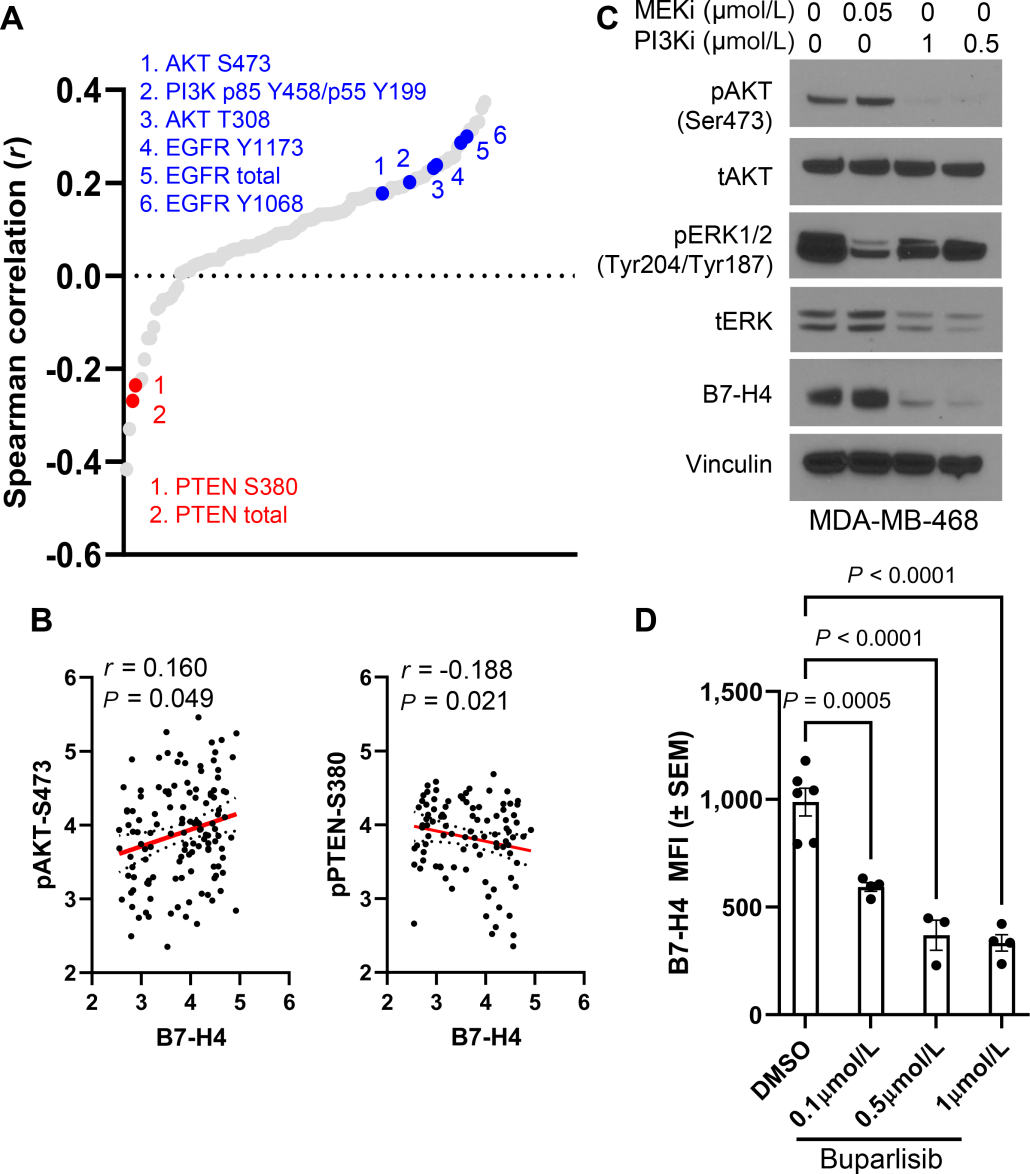
B7-H4 expression is regulated by PI3K signaling. **A,** Spearman correlations in protein expression from RPPA data collected from patients from the ISPY2 trial. Key positive correlations are called out in blue and key negative correlations are called out in red. **B,** RPPA data show strong positive correlations between B7-H4 and AKT (pAKT-Ser 473) and strong negative correlations between B7-H4 and PTEN (Ser 380) regardless of tumor hormone receptor status. **C,** PI3K inhibitor (buparlisib) treatment for 72 hours robustly decreases B7-H4 expression in human MDA-MB-468 breast cancer cells, while MEK inhibitor (trametinib) has no effect. **D,** Likewise, in MMTV-neu cells, buparlisib reduces B7-H4 expression in dose-dependent manner after 72 hours. Data were analyzed by one-way ANOVA with Dunnet *post hoc* test for multiple comparisons, *P* < 0.0001 and *P* = 0.0005).

To determine other possible pathways regulating B7-H4 expression in tumor cells, we utilized published data from the I-SPY2 neoadjuvant clinical trial of early-stage breast cancer at high risk of recurrence (NCT01042379) that were assayed with RPPA from laser-capture microdissected tumor regions (43, 18). These data include measurements of 121 protein/phosphoproteins in 151 patients treated with NAC alone or NAC + pembrolizumab, with associated clinical outcomes data. For this study, additional RPPA measurements using the same lysates were made for B7-H4 expression in the tumor compartment and compared with the existing phosphoproteomic data. We tested for the existence of significant positive or negative correlations between B7-H4 protein expression and additional tumor proteins from this cohort (Fig. 3A). Interestingly, we observed strong positive correlations of B7-H4 with PI3K and pAKT (as well as EGFR, which can activate PI3K) signaling on tumor cells, but negative correlations between B7-H4 and PTEN expression, a negative regulator of PI3K activity (Fig. 3A and B). On the basis of these findings, we tested whether specific inhibition of the PI3K pathway affected B7-H4 expression in breast cancer cells. MDA-MB-468 cells are a basal human TNBC cell line with endogenous B7-H4 expression (Fig. 2D). When these cells were treated with a pan-PI3K inhibitor (buparlisib) for 72 hours, B7-H4 expression was ablated (Fig. 3C). We also tested the effect of the same pan-PI3K inhibitor in murine MMTV-neu epithelial cells that as shown above also have high levels of endogenous B7-H4. Like MDA-MB-468 cells, surface B7-H4 expression decreased on the MMTV-neu epithelial cells in a concentration-dependent manner when measured by flow cytometry (Fig. 3D). Taken together, these data elucidate a potential mechanism of B7-H4 regulation by PI3K signaling in breast tumors.

### B7-H4 Expression Induces Moderate Resistance to Single-agent Anti-PD-L1 Immunotherapy in Mice

**FIGURE 4:**
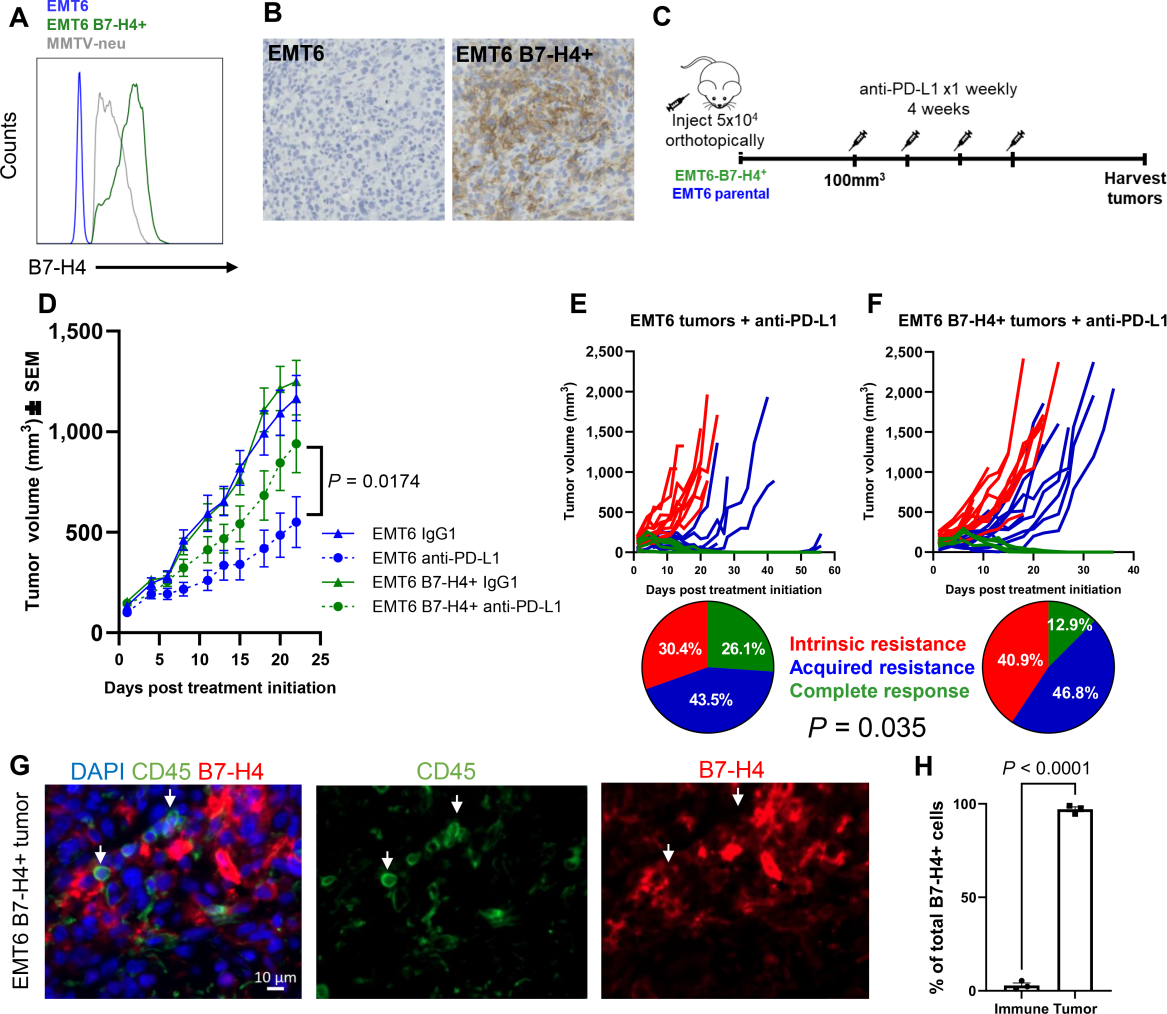
Overexpression of B7-H4 in the EMT6 murine model induced resistance to anti-PD-L1 ICI. **A,** EMT6 cells were virally transduced with the pBabe-B7-H4 retroviral vector and overexpress exogenous murine B7-H4. **B,** EMT6 B7-H4+ tumors maintain high B7-H4 expression *in vivo* assessed by IHC. **C,** Animals were orthotopically injected with EMT6 cells ± B7-H4 and treated 1x per week with anti-PD-L1 at 200 µg (first dose) or 100 µg (subsequent doses) for 4 weeks, after tumors reached 100 mm^3^. **D,** EMT6-B7-H4^+^ tumors are significantly resistant to anti-PD-L1 immunotherapy compared with control tumors. Data were analyzed by one-way ANOVA of individual AUC values with Tukey *post hoc* test for multiple comparisons between EMT6 anti-PD-L1 and EMT6-B7-H4^+^ anti-PD-L1–treated groups (*P* = 0.0174, EMT6 Isotype *n* = 21, EMT6 anti-PD-L1 *n* = 23, EMT6-B7-H4^+^ Isotype *n* = 21, EMT6-B7-H4^+^ anti-PD-L1 *n* = 23. Data were collected from a total of three independent experiments). **E** and **F,** When tumor response is categorized into three groups, EMT6-B7-H4^+^ tumors have overall greater intrinsic resistance to treatment and reduced complete response compared with EMT6 control tumors (*P* = 0.035, *χ*^2^ = 6.683, df = 2, *n* = 23 mice for EMT6 parental tumors and *n* = 31 mice for B7-H4 tumors). **G,** EMT6-B7-H4^+^ tumors were stained by mIF. B7-H4 is expressed on CD45^−^ tumor cells *in vivo*. Representative image shown. Scale bar 10 µm. **H,** Quantification of **G**, *n* = 3 mice. Data analyzed by unpaired *t* test. Data were analyzed in GraphPad Prism v10.

Currently, patients with TNBC (early stage and advanced) are eligible for pembrolizumab therapy (7, 12). We wanted to assess whether B7-H4 was acting as a mechanism of tumor resistance to ICIs, specifically the anti-PD-1/L1 axis, and could be a potential biomarker of a lack of patient response to ICIs. We overexpressed murine B7-H4 in EMT6 cells, a mesenchymal basal-like murine model that does not express B7-H4 (Fig. 2C and Fig. 4A). Compared with MMTV-neu cells that endogenously express B7-H4, the level of enforced expression is slightly higher in this tumor model. These tumors maintain high levels of B7-H4 *in vivo* (Fig. 4B). As previously shown, EMT6 tumors are sensitive to treatment with anti-PD-L1 (59). We treated EMT6-B7-H4^+^ and parental (vector alone control) tumors with anti-PD-L1 (Genentech, Clone 6E11; Fig. 4C). Compared with parental EMT6 controls, EMT6-B7-H4^+^ tumors had moderate resistance to anti-PD-L1 treatment (Fig. 4D). This model is heterogeneously responsive to anti-PD-L1 immunotherapy and even systematically treated, genetically matched mice can demonstrate intrinsic resistance, acquired resistance, or complete response, classified on the basis of the tumor growth curves (Fig. 4E). Fewer mice with EMT6-B7-H4^+^ tumors completely cleared their tumors and more mice had intrinsic resistance compared with the EMT6 control cohort (Fig. 4F). B7-H4 has also been reported to be expressed on some macrophage populations (60–63). We stained tumor sections by mIF to identify CD45^+^ and B7-H4^+^. We found (CD45^−^) tumor cells made up nearly all B7-H4^+^ cells *in vivo* (Fig. 4G and H). We found no additional B7-H4^+^ CD45^+^ immune cells in additional organs in the BALB/c mouse (Supplementary Fig. S3A). Interestingly, we did observe CD45^+^ B7-H4^+^ cells in C57BL/6 spleens and intestine (Supplementary Fig. S3A–S3D). On the basis of morphologic phenotype and the location within the tissue, these are likely B7-H4^+^ macrophages. Together, these data suggest B7-H4 tumor cell expression in EMT6 tumors contributes to immunotherapy resistance by altering tumor susceptibility to ICI, and as a side observation, notes a possible and interesting strain-specific difference in B7-H4 expression between BALB/c and C57BL/6 mice which could be important to others in the field for future mechanistic studies in preclinical models.

### Anti-PD-L1 Treatment did not Induce a Proinflammatory Immune Response in B7-H4± Tumors

**FIGURE 5:**
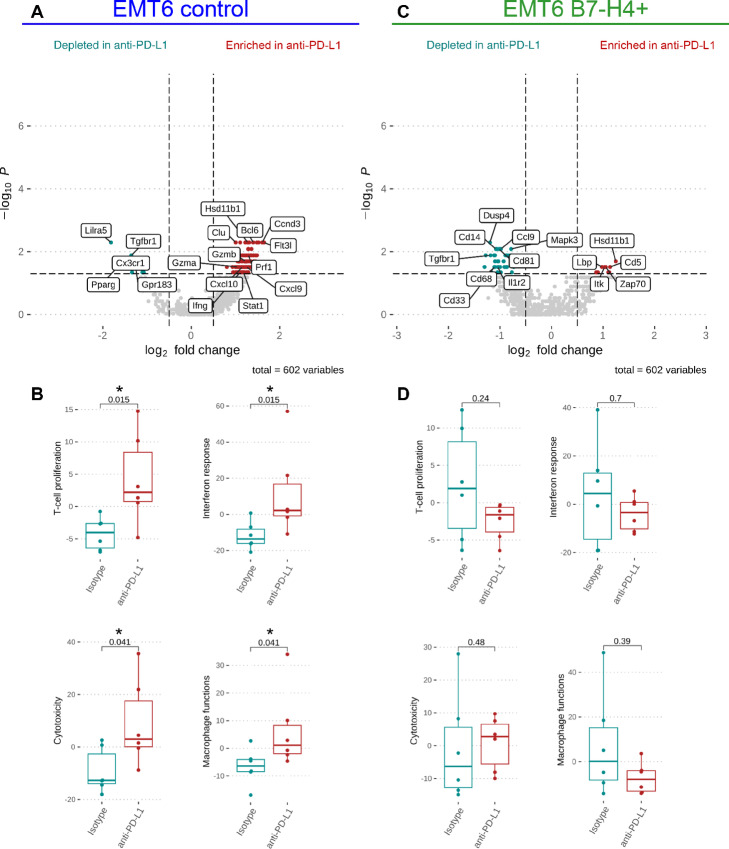
Anti-PD-L1 did not induce a proinflammatory immune response in B7-H4^+^ tumors. CD45^+^ cells were sorted from EMT6 tumors ± B7-H4 and subjected to NanoString gene expression analysis using the Mouse Pan-Cancer Immune Panel. **A,** Differentially expressed genes from CD45^+^ sorted cells from EMT6 control tumors when treated with anti-PD-L1 or isotype control and harvested 7 days after treatment. **B,** Changes in immune gene sets between isotype and anti-PD-L1–treated tumors. **C,** Differentially expressed genes from CD45^+^ sorted cells from EMT6-B7-H4^+^ tumors when treated with anti-PD-L1 or isotype control and harvested 7 days after treatment. **D,** Changes in immune gene sets between treatment groups of EMT6-B7-H4^+^ tumors. Data were analyzed by Wilcoxon rank-sum test. Genes with log_2_ fold change >0.5 or <−0.5 and *P*-value <0.01 were regarded as significant. *n* = 6 mice per group for all groups.

B7-H4 is more highly expressed in immune cold human breast tumors, and B7-H4^+^ tumors in mice were less responsive to anti-PD-L1 therapy. Therefore, we asked how the amount and functional status of TILs and myeloid cells were impacted by B7-H4 expression with or without treatment with ICI. When we assessed infiltrating immune cells (CD45^+^) in our tumor model, we found similar populations of T cells and myeloid cells regardless of B7-H4 status (Supplementary Fig. S4). We next performed NanoString gene expression analysis using the Mouse Pan-Cancer Immune Panel of 770 genes to identify markers of functional changes in the tumor immune microenvironment. We wanted to test whether B7-H4 exerts an immunosuppressive effect in the context of immunotherapy-induced activation that could explain the lack of response to anti-PD-L1 in our tumor model. We compared sorted CD45^+^ tumor immune cells between EMT6 tumors with and without B7-H4 overexpression 7 days post-treatment with anti-PD-L1. In the EMT6 control tumors, we saw an increase in transcriptomic markers of immune cell activation after anti-PD-L1 treatment compared with isotype-treated tumors (Fig. 5A). Many of these proinflammatory genes are expected in antitumor immunity including *Gzma*, *Gzmb*, *Prf1*, *Ifng*, and *Cxcl9/10* (Supplementary Table S1). We further compared functional immune gene sets and observed markers of an immune-activated environment after anti-PD-L1 treatment (Fig. 5B). In contrast, B7-H4^+^ tumors did not have the same markers of immune activation with anti-PD-L1 treatment (Fig. 5C and D). While there are some markers of T-cell activity including *Zap70* and *Lck*, these samples lack the upregulation of proinflammatory genes found in the EMT6-treated tumors (Supplementary Table S2). In addition, B7-H4+ tumors in the isotype group have high expression of immunosuppressive genes including *Tgfbr1* (TGFβ receptor), *Cd33*, and *CD68* (tumor-associated macrophage markers). These data suggest B7-H4 is functioning to inhibit full immune activation following ICI and associated with an immunosuppressive gene signature in the EMT6-B7-H4^+^ model.

We were also interested in whether CD45^+^ immune cells expressed markers of immune-activated status without ICI treatment. We measured gene expression in the CD45^+^ cells of early-stage, isotype-treated tumors ± B7-H4 expression (harvested 7 days after treatment). Genes involved in macrophage function were elevated in B7-H4^+^ tumors, but there were no other significantly different genes (Supplementary Fig. S5A and S5B). In CD45^+^ cells of later stage tumors (harvested at 500 mm^3^), genes involved in macrophage function were still elevated in B7-H4^+^ tumors (Supplementary Fig. S5C). There was also a trend toward decreased cytotoxicity gene expression in the immune compartment that was not observed at the earlier timepoint (Supplementary Fig. S5D). *Mrc1*, a marker of M2 macrophages, was more highly expressed in B7-H4^+^ tumors, suggesting the elevated macrophage function could be immunosuppressive (Supplementary Fig. S5C). A full list of differentially expressed genes, NanoString published gene set lists, and gene expression data are included (Supplementary Tables S1–S6). Together, these data suggest B7-H4 is contributing to an immunosuppressive immune microenvironment and is inhibiting immune activation after treatment with anti-PD-L1.

### B7-H4 Expression Does not Contribute to Immunotherapy Resistance in Human Breast Cancers

**FIGURE 6:**
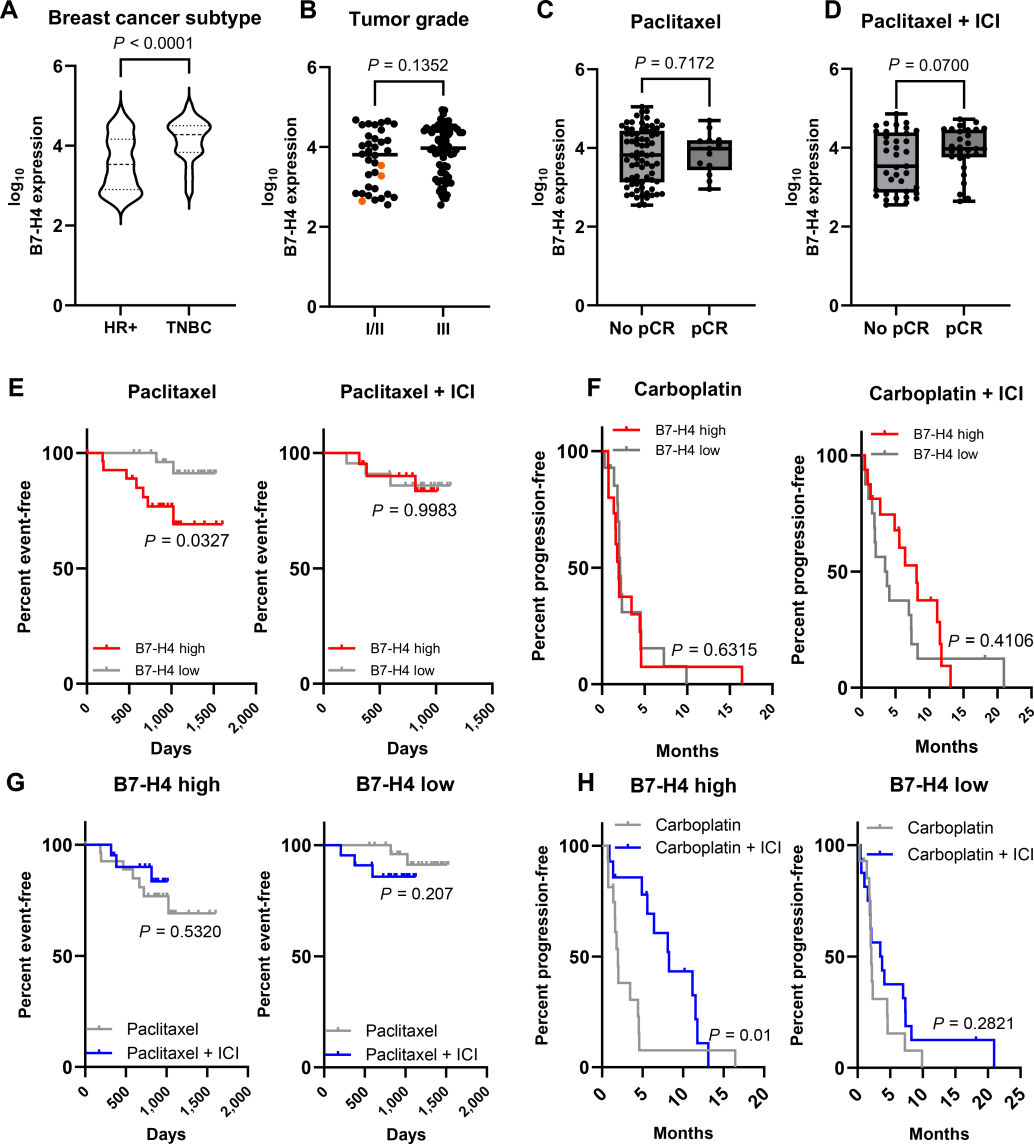
B7-H4 expression does not correlate with resistance to chemotherapy + immunotherapy in human breast tumors. Patients were from the I-SPY2 clinical trial (paclitaxel control and pembrolizumab arms) or the TBCRC 043 clinical trial (carboplatin control and atezolizumab arms). **A,** In breast tumors from the I-SPY2 clinical trial (control and pembrolizumab arms), B7-H4 expression is higher in TNBC tumors compared with HR+ tumors. Data were analyzed by unpaired *t* test. **B,** In the same patient cohort, B7-H4 expression is not higher in grade III tumors compared with grade I (orange dots) or II. Data analyzed by unpaired *t* test. **C** and **D,** B7-H4 expression is not correlated with pCR in tumors regardless of HR status, treated with either paclitaxel or paclitaxel + pembrolizumab (ICI). Data analyzed by unpaired *t* test. **E,** EFS in HR^+^ (*n* = 89) and TNBC (*n* = 62) tumors from the I-SPY2 cohort. Tumors with high B7-H4+ expression (top 33% of patients) have worse EFS when treated with paclitaxel alone and no survival benefit when treated with paclitaxel + ICI. Data were analyzed by log-rank Mantel–Cox test. **F,** In the metastatic setting, PFS stratified by B7-H4 expression (top and bottom 33% of cohort) from primary breast biopsy or metastatic lesion in patients from the TBCRC 043 trial does not correlate with B7-H4 expression in either control or carboplatin + atezolizumab (ICI) groups. Data were analyzed by log-rank Mantel–Cox test. **G** and **H,** We also assessed survival by treatment status. Metastatic tumors (H) from TBCRC 043 with high B7-H4 expression had significantly improved PFS to ICI, and nonmetastatic tumors (I-SPY2) had minimal improvement to ICI (G). Data were analyzed by log-rank Mantel–Cox test. *n* = 151 patients for A–E and G; *n* = 91 patients for F and H.

Patients with breast cancer with early-stage (II–III) and advanced (PD-L1^+^) TNBC receive chemotherapy with pembrolizumab as standard of care. We tested whether B7-H4 expression in these patient populations also associated with ICI resistance. In the I-SPY2 RPPA cohort (NCT01042379) receiving paclitaxel ± pembrolizumab (followed by doxorubicin and cyclophosphamide), we observed, as others have shown (29, 64), that B7-H4 expression was higher in TNBC tumors compared with HR+ tumors, but was expressed in HR+ tumors (Fig. 6A). B7-H4 expression did not correlate with tumor grade (Fig. 6B). We also observed no correlation with B7-H4 expression and pathologic complete response (pCR) regardless of treatment with paclitaxel alone or paclitaxel plus pembrolizumab (Fig. 6C and D). We wanted to test for any association with B7-H4 expression and patient survival, to see whether the human data recapitulated our preclinical murine model. To that end, we analyzed both the patients with early-stage breast cancer (from ISPY2/NCT01042379) and advanced, metastatic TNBC (from NCT03206203). The patients with metastatic TNBC received carboplatin ± atezolizumab (anti-PD-L1; NCT03206203; ref. 40). When B7-H4 expression was stratified into high (top 33%) and low (bottom 33%) patient subgroups, high expression was associated with worse event-free survival (EFS) in chemotherapy-alone treated patients with early-stage breast cancer, which appeared to be overcome by anti-PD-1 combination therapy (Fig. 6E). However, when we adjusted for HR status using a Cox proportional hazards analysis, this finding was no longer significant (*P* = 0.39). We observed no correlation with progression-free survival (PFS) in patients with metastatic TNBC treated with anti-PD-L1 therapy (Fig. 6F). To ask more specifically whether B7-H4 high or low expressers differentially benefit from ICI, we compared survival by arm in each B7-H4 expression group. Paradoxically, we observed an improved benefit of B7-H4 expression with PFS after ICI in the metastatic setting, and no association with postsurgical EFS in the early setting (Fig. 6G and H). These findings deviate from our observations in the murine model, suggesting that additional complex signaling mechanisms may be altering immunotherapy response. In fact, we found different endogenous B7-H4 expression patterns even between two murine models (Supplementary Fig. S3). Collectively, these data suggest B7-H4 may not be a reliable biomarker for ICI resistance in patients with breast cancer and more research is needed to understand its regulation in human and mouse cancers.

## Discussion

We have shown that B7-H4, which is highly conserved between mice and humans (28), is strongly associated with epithelial cell status in both murine and human breast cancer cells and is regulated in part by PI3K pathway activity. However, that may be where the similarities end. In our EMT6 murine model, B7-H4 expression contributed to single-agent immunotherapy resistance and decreased immune cell function (particularly T-cell function, as has been described previously; refs. 27, 28, 30). In addition, in a preclinical murine C3TAg tumor model, Liu and colleagues described murine B7-H4 knockout (KO) was sufficient to sensitize tumors to immunotherapy (65). Surprisingly, when we assessed patients with early-stage and advanced breast cancer, we found the opposite phenomenon. B7-H4 expression had minimal effect to ICI response and in one cohort was even associated with improved survival. The biggest difference in study design between the clinical trials and our preclinical models was the inclusion of chemotherapy with the immunotherapy regimen. The patients analyzed from NCT01042379 had early-stage breast cancer and received neoadjuvant paclitaxel with four rounds of pembrolizumab, followed by doxorubicin and cyclophosphamide (18). The patients as part of NCT03206203 had metastatic TNBC and received carboplatin and atezolizumab together intravenously every 3 weeks until intolerability (20). When we combined anti-PD-L1 with chemotherapy in our preclinical EMT6 model ± B7-H4, we observed no tumor response to paclitaxel chemotherapy alone and no improved tumor response with paclitaxel + anti-PD-L1 over anti-PD-L1 alone (Supplementary Fig. S6). This suggests different mechanisms of action between the human and mouse tumor response to chemotherapy and immunotherapy.

The correlation between B7-H4 and epithelial cell status and related transcription factors and the regulation by PI3K signaling in cancer cells suggests a potential novel mechanism for B7-H4 regulation, distinct from PD-L1, to which the ligand is commonly compared. In contrast to others’ findings, we observed no inverse correlation between B7-H4 and PD-L1 expression within tumor cell regions (Supplementary Fig. S7), but this does not rule out different regulatory mechanisms. It does, however, rule out any suppressive effect of PD-L1 on B7-H4 within the same tumor cell. Instead, PD-L1^+^ tumors tend to be more inflamed than B7-H4+ tumors, and PD-L1 can often be expressed on immune cells within the tumor stroma. Therefore, between breast tumors, there may be a preference for PD-L1 expression over B7-H4 expression indicated by the immune infiltration or lack thereof, but within tumors or tumor cells, there is no inhibitory effect of the two checkpoint ligands that we observed (i.e., no direct reciprocal regulation).

In our preclinical model, B7-H4 had a moderate effect on immune cell signaling, most notably in a reduction of cytotoxic T-cell function and an increase of immunosuppressive macrophage function, assessed by NanoString gene expression. Interestingly, B7-H4 expression in treated tumors seems to dampen or inhibit the same induction of immune activation by anti-PD-L1 treatment in the EMT6 controls. Identifying the mechanism(s) of B7-H4–mediated immunosuppression within a complex tumor microenvironment, including identifying the receptor and cells expressing the receptor, is an avenue for future experiments.

There are several limitations and caveats to our study. First, we demonstrated B7-H4–induced anti-PD-L1 resistance (gain of function; sufficiency) in a single mouse model. Moreover, we were unable to identify a reciprocal loss-of-function model (i.e., B7-H4-KO) to test necessity of B7-H4 expression for anti-PD-L1 resistance; however, given that breaks in the tumor immunity cycle can exist at nearly any point in the path, identifying a model that innately expresses B7-H4 in the tumor compartment, and wherein this feature is the sole effector of resistance to anti-PD-L1 is far less likely given the general paucity of models in the field. Nonetheless, independent and external confirmation was recently published by Liu and colleagues, suggesting broader applicability and validity in murine breast tumors, including loss of function leading to enhanced sensitivity to anti-PD-L1 (65).

We also observed resistance to chemotherapy in the EMT6 model regardless of B7-H4 status, prohibiting a more direct comparison in study design with the human clinical trial data. In addition, we observed changes in immune cell gene expression with tumor B7-H4 expression that were not supported by our flow cytometry experiments. These contrasting findings could be due to differences in phenotyping based on gene expression profiling (more quantitative, and reliable but less functional) versus phenotyping by several limited characteristic markers like CD206 expression or granzyme staining. Nonetheless, the combined analysis of both mRNA profiling and immunophenotyping by flow suggest changes in macrophage functionality and generally less T-cell activation with B7-H4 expression, particularly in later tumor stages. Future experiments using detailed phenotyping flow cytometry as well as RNA sequencing may shed more light on the mechanism of B7-H4 immunosuppression *in vivo*.

The patients with early-stage breast cancer were also a mixed cohort with HR^+^ and TNBC and were combined for analysis due to sample size constraints and because both groups demonstrated considerable, but heterogeneous B7-H4 expression. TNBC may have higher expression of B7-H4, but it is not exclusive to that subtype and could be highly expressed in immune-cold tumors regardless of subtype. For example, the MMTV-neu murine model emulates luminal-like HER2^+^ breast cancer and endogenously expresses B7-H4.

In conclusion, our data show a broad exploration of B7-H4 expression and function in murine and human breast cancer. On the basis of the difference in tumor progression, or lack thereof, in the human cohorts and mouse models, future understanding of the mechanisms of B7-H4 *in vivo* are essential to rule out or include B7-H4 as a potential biomarker for future patients with breast cancer. Instead of an immune checkpoint, B7-H4 could be a better target for antibody–drug conjugate (ADC) development, as multiple companies are doing (64, 66). In fact, to our knowledge, there are no B7-H4 blocking antibodies in clinical trials. These ADCs target B7-H4 independent of ICI resistance and may prove a better direction for the field of breast cancer treatment.

## Supplementary Material

Figure S1 Supplemental Figure 1. MMTV-neu epithelial and mesenchymal cells did not undergo EMT or MET. Single cell-derived clones were isolated from parental, heterogeneous MMTV-neu cells by FACS single-cell limiting dilutions. 16 epithelial and 10 mesenchymal single-cell clones were passaged independently for over 20 passages. After 13 passages, conditioned media from the alternate cell line was collected, filtered, and applied to a passage of each single-cell clone. Independent clones or clones treated with conditioned media did not undergo epithelial-to-mesenchymal or mesenchymal-to-epithelial transition in vitro. Two representative clones from each cell line are shown above.

Figure S2 Supplemental Figure 2. B7-H4 expression is not affected by type I or II interferon or TGF-β treatment in vitro. MMTV-neu epithelial cells that have high levels of endogenous B7-H4 were treated for 72 hours with IFNα or IFNγ at 100ng/mL. B7-H4 expression was analyzed by flow cytometry (n=4-5 per group). Similarly, B7-H4 expression was not altered by TGF-β expression (10 ng/mL) in vitro after 72 hours. Data were analyzed by One-way ANOVA or unpaired t-test.

Figure S3 Supplemental Figure 3. B7-H4 was expressed on some tissue immune cells in the C57BL/6 model, but not the BALB/c model. (A) Formalin-fixed, paraffin-embedded sections of BALB/c and C57BL/6 mice were stained for B7-H4, CD45, and DAPI using multiplexed immunofluorescence, or B7-H4 by IHC. CD45+ B7-H4+ cells were observed in spleen and intestine in C57BL/6 mice but not BALB/c mice. (B) Based on morphological characterization, these are likely macrophages. BALB/c spleen, intestine, and other healthy tissues examined (lymph node, fat pad, lung) had no B7-H4+ immune cells. Scale bar 20µm. (C) Flow cytometry of B7-H4+ cells are not present in the BALB/c spleen but are in the C57BL/6 spleen, similar to our findings by mIF. (D) Representation of flow cytometry scatter plots. B7-H4+ immune cells were only found in the C57BL/6 spleen, not bone marrow (b.m.)

Figure S4 Supplemental Figure 4. B7-H4 expression did not change quantity of infiltrating tumor immune cells in vivo regardless of anti-PD-L1 treatment. Untreated and anti-PD-L1 treated EMT6 tumors ± B7-H4 were dissociated to single cell suspension and subjected to flow cytometry with a 14 (for myeloid cells) or 17 (for T cells) color panel on a CyTEK Aurora. n = 3/group for control and 8/group for treated samples. Data were analyzed by One-way ANOVA with Sidak’s post-hoc test for multiple comparisons between the EMT6 control anti-PD-L1 and EMT6 B7-H4+ anti-PD-L1 treatment groups. One-way ANOVA was not significant between groups. Data were analyzed in GraphPad Prism v10.

Figure S5 Supplemental Figure 5. CD45+ gene expression changes from early and later tumor stage between EMT6 control and B7-H4+ tumors. (A) CD45+ cells were isolated from EMT6 control or B7-H4+ tumors 7 days post treatment with isotype control antibody. Differentially expressed genes are shown. n = 6 mice per group. (B) Macrophage function was upregulated in B7-H4+ tumors, but minimal other differences were seen. (C) Differentially expressed genes between control and B7-H4+ tumors at a later stage. n = 12 mice per group. CD45+ cells were harvested when tumors reached 500mm3. (D) Macrophage function remains elevated in B7-H4+ tumors, but minimal differences were detected. Data were analyzed by Wilcoxon rank sum test. Genes with log2 fold change >0.5 or <-0.5 and p-value <0.01 were regarded as significant.

Figure S6 Supplemental Figure 6. EMT6 tumors do not respond to single-agent chemotherapy. (A) EMT6 parental or B7-H4+ tumors treated with vehicle (isotype control), anti-PD-L1, paclitaxel chemotherapy, or anti-PD-L1 + paclitaxel (n=15/group parental and n = 10/group B7-H4+). We observed no tumor response to paclitaxel single-agent therapy and thus the response observed in the combination treatment group is driven by anti-PD-L1 effects. (One-way ANOVA with Tukey’s post-hoc test for multiple comparisons. P values as shown. (B) Survival of EMT6 parental or B7-H4+ tumors. We observed significant survival of the anti-PD-L1 and combination treatment groups in both tumor types compared to vehicle or paclitaxel treatment groups. (Data were analyzed by Log-rank Mantel Cox test. Statistics performed in GraphPad Prism v10).

Figure S7 Supplemental Figure 7. B7-H4 and PD-L1 are not mutually exclusive on tumor cells. Data shown are reverse phase protein array expression data of B7-H4 and PD-L1 (SP142, 22C3, or Atezolizumab) from the I-SPY2 patient cohort. Samples were a mixture of TNBC and ER+ tumors and were treated with chemotherapy ± anti-PD-1. Depending on the antibody clone selected to detect PD-L1 expression, B7-H4 had no correlation, or a positive correlation to PD-L1 expression in these tumors. Data analyzed by spearman correlation and shown with linear regression best-fit line. n = 151 patients.

Table S1 Supplementary Table 1. DE genes between isotype treated and anti-PD-L1 treated EMT6 tumors.

Table S2 Supplementary Table 2. DE genes between isotype treated and anti-PD-L1 treated EMT6-B7-H4+ tumors.

Table S3 Supplementary Table 3. DE genes between isotype treated EMT6 and EMT6-B7-H4+ tumors, early timepoint, 7 days post isotype treatment.

Table S4 Supplementary Table 4. DE genes between EMT6 and EMT6-B7-H4+ tumors harvested at 500mm^3.

Table S5 Supplementary Table 5. Nanostring gene set gene list included in manuscript analysis.

Table S6 Nanostring gene expression data related to Figure 5 and Figure S5 with related metatdata tables.
