# Supplementary material for: Epithelial Expressed B7-H4 Drives Differential Immunotherapy Response in Murine and Human Breast Cancer
Source: Cancer Res Commun. 2024 Apr 24;4(4):1120–34. doi: 10.1158/2767-9764.CRC-23-0468 (PMC11041871; doi:10.1158/2767-9764.CRC-23-0468)
Supplement: Figure S1 — Supplemental Figure 1. MMTV-neu epithelial and mesenchymal cells did not undergo EMT or MET. Single cell-derived clones were isolated from parental, heterogeneous MMTV-neu cells by FACS single-cell limiting dilutions. 16 epithelial and 10 mesenchymal single-cell clones were passaged independently for over 20 passages. After 13 passages, conditioned media from the alternate cell line was collected, filtered, and applied to a passage of each single-cell clone. Independent clones or clones treated with conditioned media did not undergo epithelial-to-mesenchymal or mesenchymal-to-epithelial transition in vitro. Two representative clones from each cell line are shown above. [file crc-23-0468-s01.pdf]

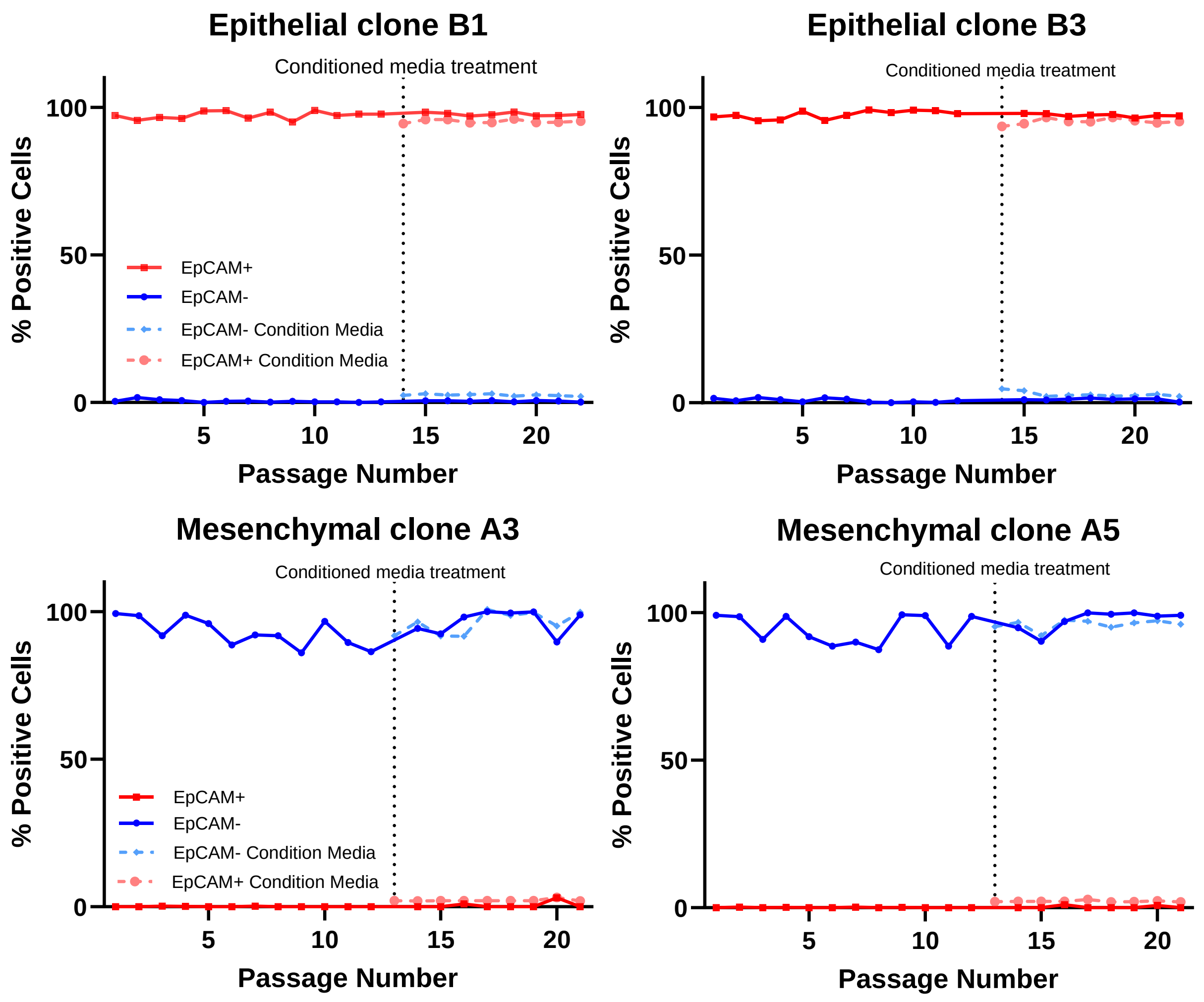

Supplemental Figure 1. MMTV-neu epithelial and mesenchymal cells did not undergo EMT or MET. Single cell-derived clones were isolated from parental, heterogeneous MMTV-neu cells by FACS single-cell limiting dilutions. 16 epithelial and 10 mesenchymal single-cell clones were passaged independently for over 20 passages. After 13 passages, conditioned media from the alternate cell line was collected, filtered, and applied to a passage of each single-cell clone. Independent clones or clones treated with conditioned media did not undergo epithelial-to-mesenchymal or mesenchymal-to-epithelial transition *in vitro*. Two representative clones from each cell line are shown above.
