# Supplementary material for: Epithelial Expressed B7-H4 Drives Differential Immunotherapy Response in Murine and Human Breast Cancer
Source: Cancer Res Commun. 2024 Apr 24;4(4):1120–34. doi: 10.1158/2767-9764.CRC-23-0468 (PMC11041871; doi:10.1158/2767-9764.CRC-23-0468)
Supplement: Figure S2 — Supplemental Figure 2. B7-H4 expression is not affected by type I or II interferon or TGF-β treatment in vitro. MMTV-neu epithelial cells that have high levels of endogenous B7-H4 were treated for 72 hours with IFNα or IFNγ at 100ng/mL. B7-H4 expression was analyzed by flow cytometry (n=4-5 per group). Similarly, B7-H4 expression was not altered by TGF-β expression (10 ng/mL) in vitro after 72 hours. Data were analyzed by One-way ANOVA or unpaired t-test. [file crc-23-0468-s02.pdf]

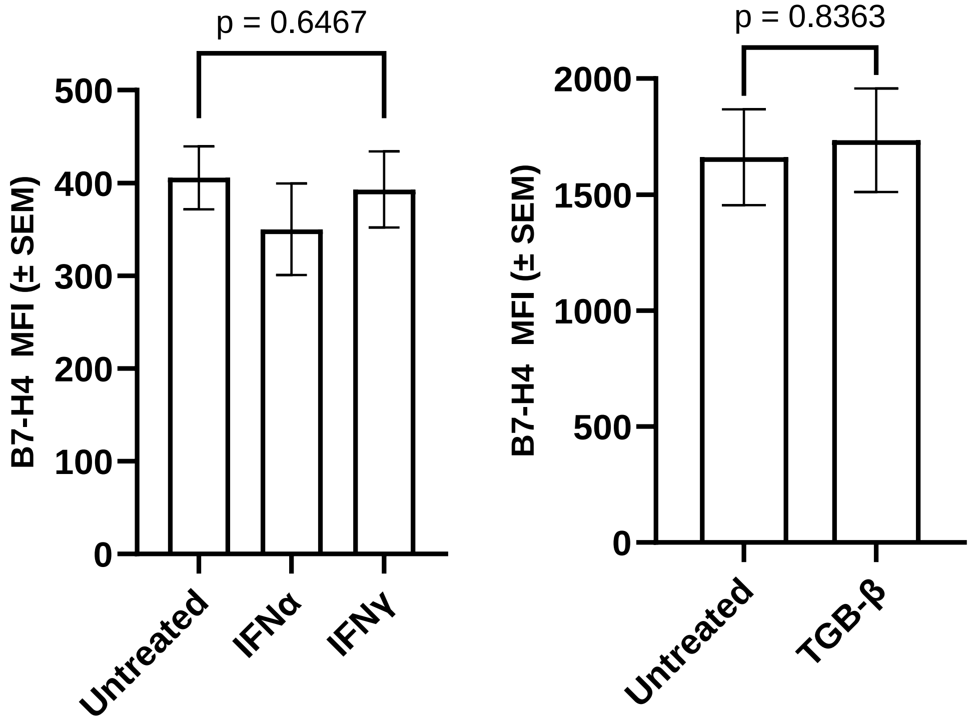

Supplemental Figure 2. B7-H4 expression is not affected by type I or II interferon or TGF-β treatment *in vitro*. MMTV-neu epithelial cells that have high levels of endogenous B7-H4 were treated for 72 hours with IFNα or IFNγ at 100ng/mL. B7-H4 expression was analyzed by flow cytometry (n=4-5 per group). Similarly, B7-H4 expression was not altered by TGF-β expression (10 ng/mL) *in vitro* after 72 hours. Data were analyzed by One-way ANOVA or unpaired t-test.
