# Supplementary material for: Epithelial Expressed B7-H4 Drives Differential Immunotherapy Response in Murine and Human Breast Cancer
Source: Cancer Res Commun. 2024 Apr 24;4(4):1120–34. doi: 10.1158/2767-9764.CRC-23-0468 (PMC11041871; doi:10.1158/2767-9764.CRC-23-0468)
Supplement: Figure S3 — Supplemental Figure 3. B7-H4 was expressed on some tissue immune cells in the C57BL/6 model, but not the BALB/c model. (A) Formalin-fixed, paraffin-embedded sections of BALB/c and C57BL/6 mice were stained for B7-H4, CD45, and DAPI using multiplexed immunofluorescence, or B7-H4 by IHC. CD45+ B7-H4+ cells were observed in spleen and intestine in C57BL/6 mice but not BALB/c mice. (B) Based on morphological characterization, these are likely macrophages. BALB/c spleen, intestine, and other healthy tissues examined (lymph node, fat pad, lung) had no B7-H4+ immune cells. Scale bar 20µm. (C) Flow cytometry of B7-H4+ cells are not present in the BALB/c spleen but are in the C57BL/6 spleen, similar to our findings by mIF. (D) Representation of flow cytometry scatter plots. B7-H4+ immune cells were only found in the C57BL/6 spleen, not bone marrow (b.m.) [file crc-23-0468-s03.pdf]

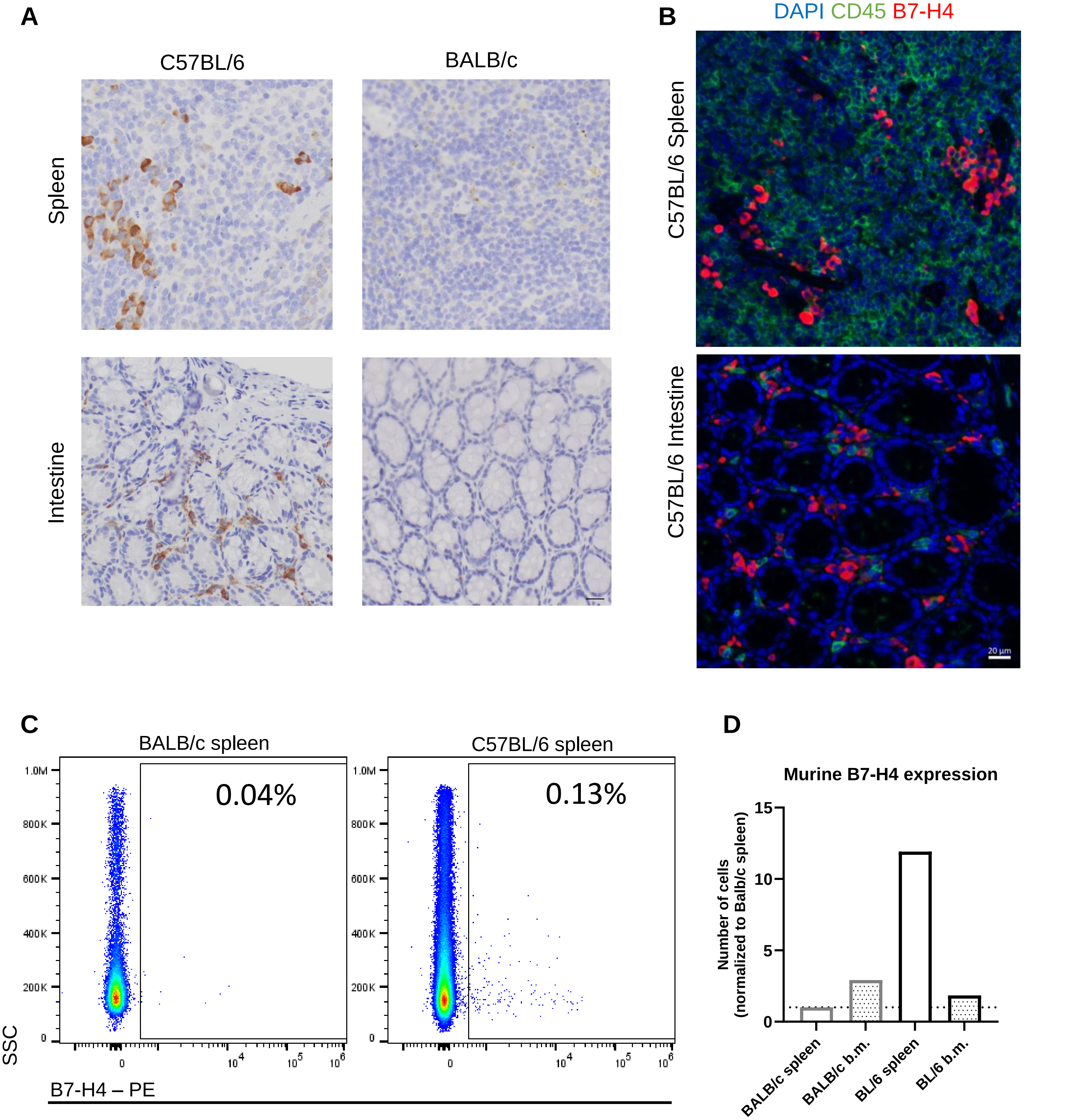

Supplemental Figure 3. B7-H4 was expressed on some tissue immune cells in the C57BL/6 model, but not the BALB/c model. **(A)** Formalin-fixed, paraffin-embedded sections of BALB/c and C57BL/6 mice were stained for B7-H4, CD45, and DAPI using multiplexed immunofluorescence, or B7-H4 by IHC. CD45+ B7-H4+ cells were observed in spleen and intestine in C57BL/6 mice but not BALB/c mice. **(B)** Based on morphological characterization, these are likely macrophages. BALB/c spleen, intestine, and other healthy tissues examined (lymph node, fat pad, lung) had no B7-H4+ immune cells. Scale bar 20µm. **(C)** Flow cytometry of B7-H4+ cells are not present in the BALB/c spleen but are in the C57BL/6 spleen, similar to our findings by mIF. **(D)** Representation of flow cytometry scatter plots. B7-H4+ immune cells were only found in the C57BL/6 spleen, not bone marrow (b.m.)
