# Supplementary material for: Epithelial Expressed B7-H4 Drives Differential Immunotherapy Response in Murine and Human Breast Cancer
Source: Cancer Res Commun. 2024 Apr 24;4(4):1120–34. doi: 10.1158/2767-9764.CRC-23-0468 (PMC11041871; doi:10.1158/2767-9764.CRC-23-0468)
Supplement: Figure S4 — Supplemental Figure 4. B7-H4 expression did not change quantity of infiltrating tumor immune cells in vivo regardless of anti-PD-L1 treatment. Untreated and anti-PD-L1 treated EMT6 tumors ± B7-H4 were dissociated to single cell suspension and subjected to flow cytometry with a 14 (for myeloid cells) or 17 (for T cells) color panel on a CyTEK Aurora. n = 3/group for control and 8/group for treated samples. Data were analyzed by One-way ANOVA with Sidak’s post-hoc test for multiple comparisons between the EMT6 control anti-PD-L1 and EMT6 B7-H4+ anti-PD-L1 treatment groups. One-way ANOVA was not significant between groups. Data were analyzed in GraphPad Prism v10. [file crc-23-0468-s04.pdf]

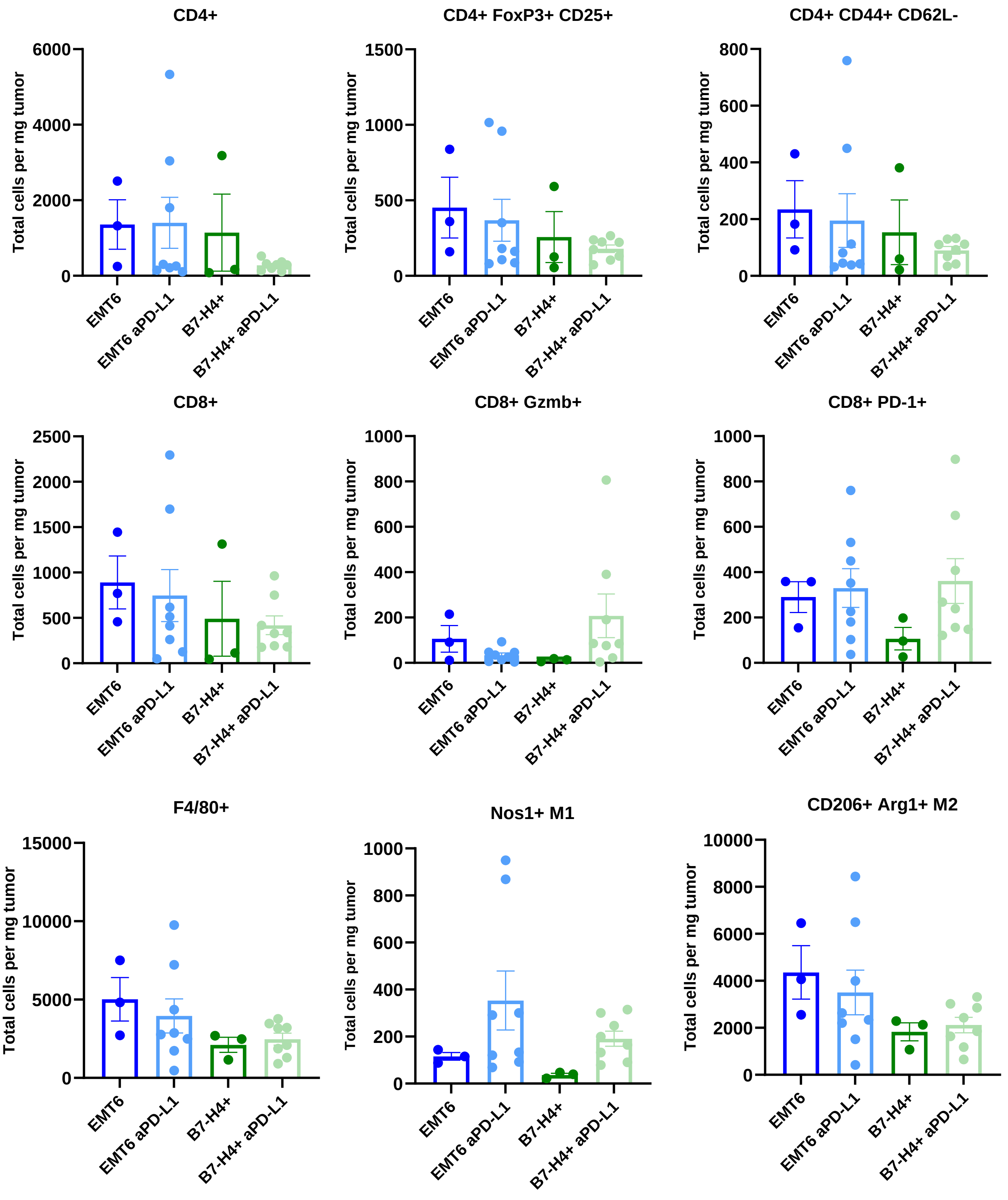

Supplemental Figure 4. B7-H4 expression did not change quantity of infiltrating tumor immune cells *in vivo* regardless of anti-PD-L1 treatment. Untreated and anti-PD-L1 treated EMT6 tumors ± B7-H4 were dissociated to single cell suspension and subjected to flow cytometry with a 14 (for myeloid cells) or 17 (for T cells) color panel on a CyTEK Aurora. n = 3/group for control and 8/group for treated samples. Data were analyzed by One-way ANOVA with Sidak's post-hoc test for multiple comparisons between the EMT6 control anti-PD-L1 and EMT6 B7-H4+ anti-PD-L1 treatment groups. One-way ANOVA was not significant between groups. Data were analyzed in GraphPad Prism v10.
