# Supplementary material for: Epithelial Expressed B7-H4 Drives Differential Immunotherapy Response in Murine and Human Breast Cancer
Source: Cancer Res Commun. 2024 Apr 24;4(4):1120–34. doi: 10.1158/2767-9764.CRC-23-0468 (PMC11041871; doi:10.1158/2767-9764.CRC-23-0468)
Supplement: Figure S5 — Supplemental Figure 5. CD45+ gene expression changes from early and later tumor stage between EMT6 control and B7-H4+ tumors. (A) CD45+ cells were isolated from EMT6 control or B7-H4+ tumors 7 days post treatment with isotype control antibody. Differentially expressed genes are shown. n = 6 mice per group. (B) Macrophage function was upregulated in B7-H4+ tumors, but minimal other differences were seen. (C) Differentially expressed genes between control and B7-H4+ tumors at a later stage. n = 12 mice per group. CD45+ cells were harvested when tumors reached 500mm3. (D) Macrophage function remains elevated in B7-H4+ tumors, but minimal differences were detected. Data were analyzed by Wilcoxon rank sum test. Genes with log2 fold change >0.5 or <-0.5 and p-value <0.01 were regarded as significant. [file crc-23-0468-s05.pdf]

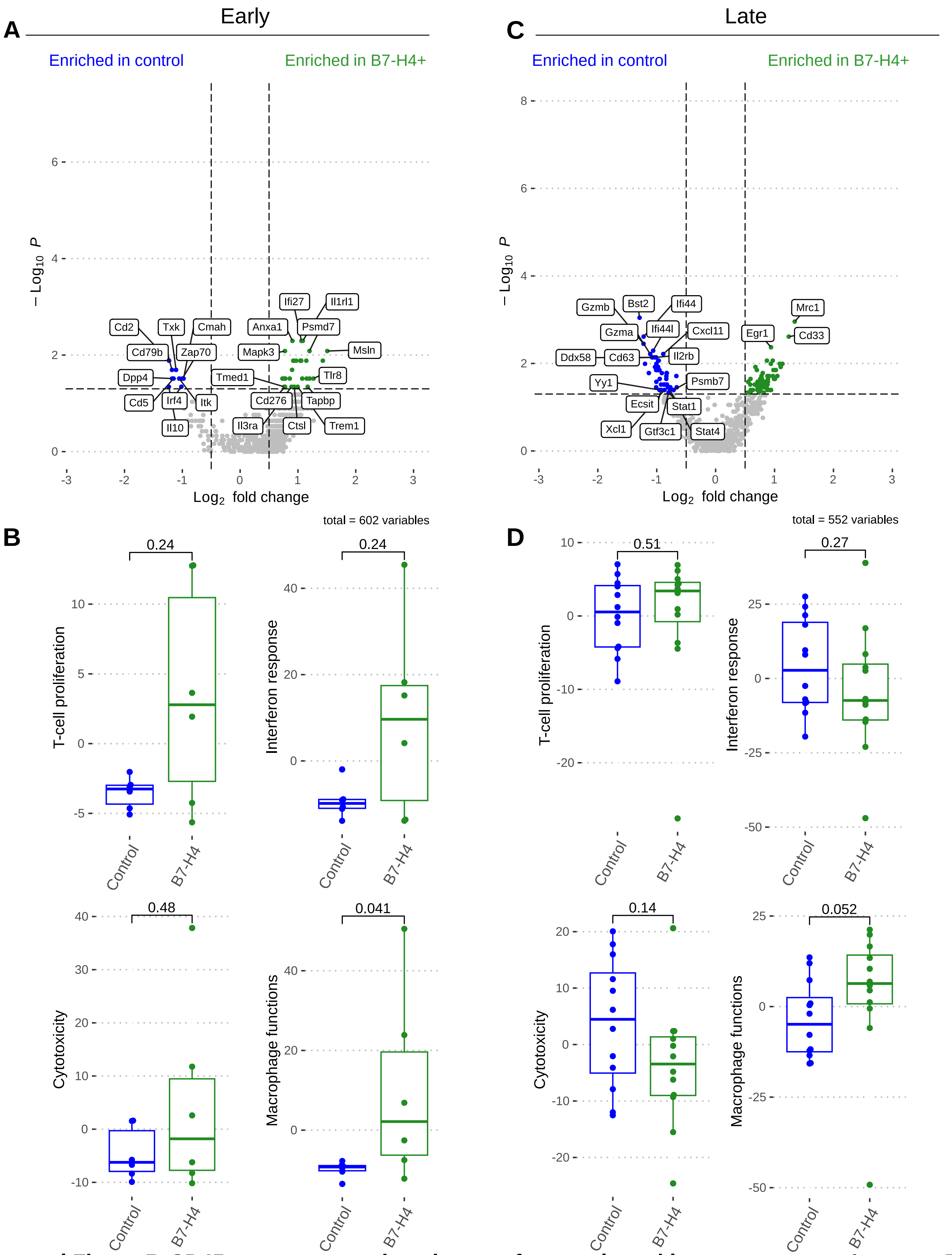

Supplemental Figure 5. CD45+ gene expression changes from early and later tumor stage between EMT6 control and B7-H4+ tumors. (A) CD45+ cells were isolated from EMT6 control or B7-H4+ tumors 7 days post treatment with isotype control antibody. Differentially expressed genes are shown. n = 6 mice per group. **(B)** Macrophage function was upregulated in B7-H4+ tumors, but minimal other differences were seen. **(C)** Differentially expressed genes between control and B7-H4+ tumors at a later stage. n = 12 mice per group. CD45+ cells were harvested when tumors reached 500mm^3. **(D)** Macrophage function remains elevated in B7-H4+ tumors, but minimal differences were detected. Data were analyzed by Wilcoxon rank sum test. Genes with log2 fold change >0.5 or <-0.5 and p-value <0.01 were regarded as significant.
