# Supplementary material for: Epithelial Expressed B7-H4 Drives Differential Immunotherapy Response in Murine and Human Breast Cancer
Source: Cancer Res Commun. 2024 Apr 24;4(4):1120–34. doi: 10.1158/2767-9764.CRC-23-0468 (PMC11041871; doi:10.1158/2767-9764.CRC-23-0468)
Supplement: Figure S6 — Supplemental Figure 6. EMT6 tumors do not respond to single-agent chemotherapy. (A) EMT6 parental or B7-H4+ tumors treated with vehicle (isotype control), anti-PD-L1, paclitaxel chemotherapy, or anti-PD-L1 + paclitaxel (n=15/group parental and n = 10/group B7-H4+). We observed no tumor response to paclitaxel single-agent therapy and thus the response observed in the combination treatment group is driven by anti-PD-L1 effects. (One-way ANOVA with Tukey’s post-hoc test for multiple comparisons. P values as shown. (B) Survival of EMT6 parental or B7-H4+ tumors. We observed significant survival of the anti-PD-L1 and combination treatment groups in both tumor types compared to vehicle or paclitaxel treatment groups. (Data were analyzed by Log-rank Mantel Cox test. Statistics performed in GraphPad Prism v10). [file crc-23-0468-s06.pdf]

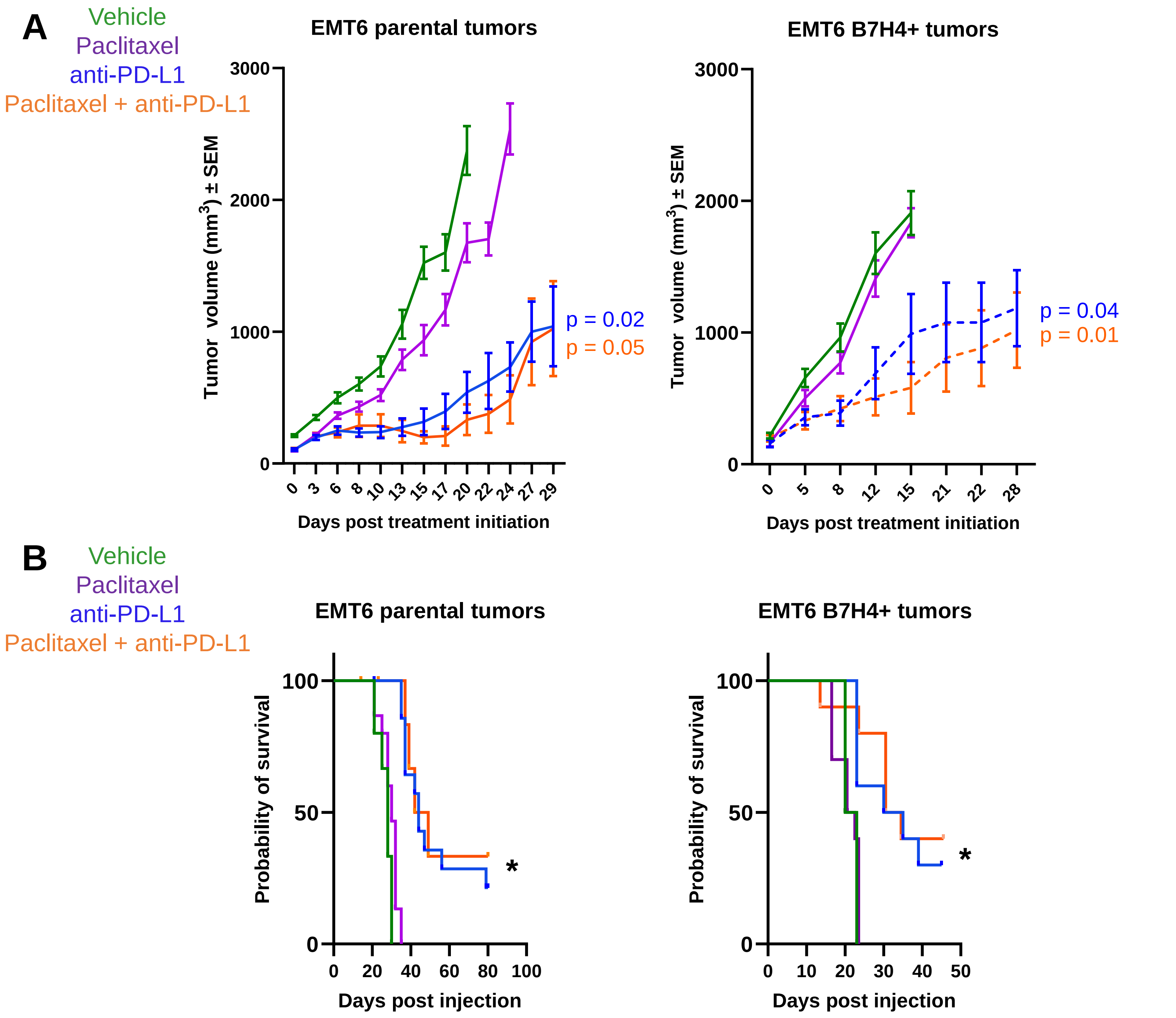

Supplemental Figure 6. EMT6 tumors do not respond to single-agent chemotherapy. (A) EMT6 parental or B7-H4+ tumors treated with vehicle (isotype control), anti-PD-L1, paclitaxel chemotherapy, or anti-PD-L1 + paclitaxel (n=15/group parental and n = 10/group B7-H4+). We observed no tumor response to paclitaxel single-agent therapy and thus the response observed in the combination treatment group is driven by anti-PD-L1 effects. (One-way ANOVA with Tukey's post-hoc test for multiple comparisons. P values as shown. **(B)** Survival of EMT6 parental or B7-H4+ tumors. We observed significant survival of the anti-PD-L1 and combination treatment groups in both tumor types compared to vehicle or paclitaxel treatment groups. (Data were analyzed by Log-rank Mantel Cox test. Statistics performed in GraphPad Prism v10).
