# Supplementary material for: Epithelial Expressed B7-H4 Drives Differential Immunotherapy Response in Murine and Human Breast Cancer
Source: Cancer Res Commun. 2024 Apr 24;4(4):1120–34. doi: 10.1158/2767-9764.CRC-23-0468 (PMC11041871; doi:10.1158/2767-9764.CRC-23-0468)
Supplement: Figure S7 — Supplemental Figure 7. B7-H4 and PD-L1 are not mutually exclusive on tumor cells. Data shown are reverse phase protein array expression data of B7-H4 and PD-L1 (SP142, 22C3, or Atezolizumab) from the I-SPY2 patient cohort. Samples were a mixture of TNBC and ER+ tumors and were treated with chemotherapy ± anti-PD-1. Depending on the antibody clone selected to detect PD-L1 expression, B7-H4 had no correlation, or a positive correlation to PD-L1 expression in these tumors. Data analyzed by spearman correlation and shown with linear regression best-fit line. n = 151 patients. [file crc-23-0468-s07.pdf]

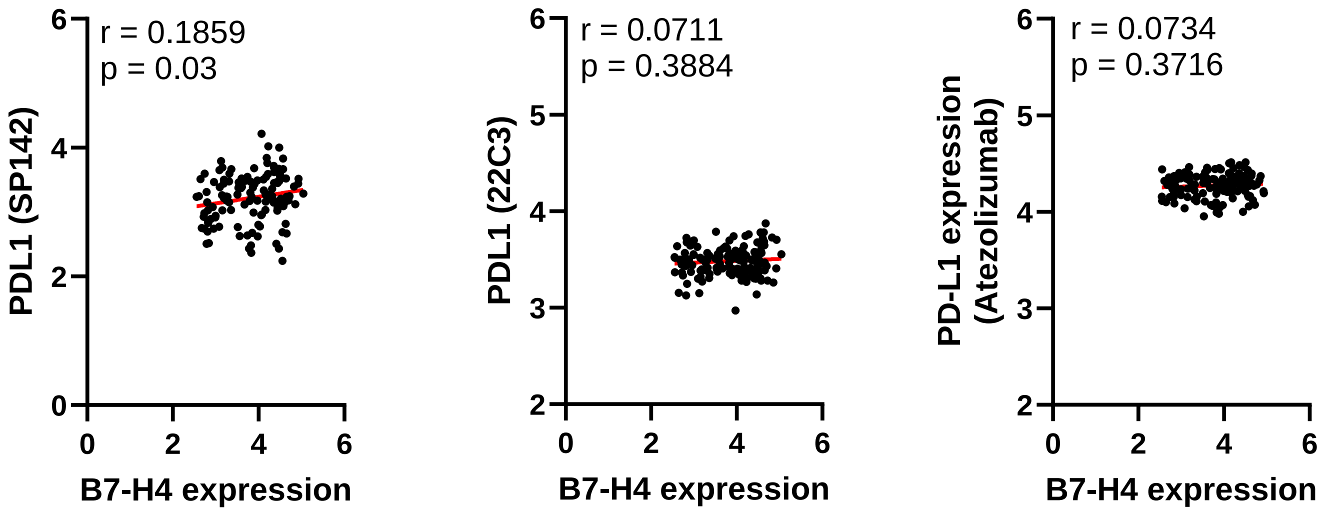

Supplemental Figure 7. B7-H4 and PD-L1 are not mutually exclusive on tumor cells. Data shown are reverse phase protein array expression data of B7-H4 and PD-L1 (SP142, 22C3, or Atezolizumab) from the I-SPY2 patient cohort. Samples were a mixture of TNBC and ER+ tumors and were treated with chemotherapy ± anti-PD-1. Depending on the antibody clone selected to detect PD-L1 expression, B7-H4 had no correlation, or a positive correlation to PD-L1 expression in these tumors. Data analyzed by spearman correlation and shown with linear regression best-fit line. n = 151 patients.
